# US expert Delphi consensus on the prevention and management of stomatitis in patients treated with datopotamab deruxtecan

**DOI:** 10.1007/s00520-025-09805-y

**Published:** 2025-08-05

**Authors:** Hope Rugo, Aditya Bardia, Debora S. Bruno, Vinicius Ernani, Erika Hamilton, Rebecca Heist, Komal Jhaveri, Benjamin Levy, Aaron Lisberg, Joyce O’Shaughnessy, Jacob Sands, Alexander Spira, Sara Tolaney, Nathaniel S. Treister, Cecile Matthews, Melissa Johnson

**Affiliations:** 1 City of Hope Comprehensive Cancer Center, Duarte, CA USA; 2 Massachusetts General Hospital, Boston, MA USA; 3 University Hospitals, Case Western Reserve University, Cleveland, OH USA; 4 Mayo Clinic, Phoenix, AZ USA; 5 Sarah Cannon Research Institute, Nashville, TN USA; 6 Memorial Sloan Kettering Cancer Institute, New York, NY USA; 7 John Hopkins Kimmel Cancer Center, Baltimore, MD USA; 8 University of California at Los Angeles, Los Angeles, CA USA; 9 Texas Oncology Baylor-Sammons Cancer Institute, Dallas, TX USA; 10 Dana-Farber Cancer Institute, Boston, MA USA; 11 Virginia Cancer Specialists Research Institute, Leesburg, VA USA; 12 Brigham and Women’s Hospital Dana-Farber Cancer Institute, Boston, MA USA; 13 Charles River Associates, Cambridge, UK

**Keywords:** Oral mucositis, Stomatitis, Adverse event management, Consensus, Dato-DXd, Dexamethasone mouthwash

## Abstract

**Background:**

Datopotamab deruxtecan (Dato-DXd) is an investigational antibody–drug conjugate that has shown promising results in phase III clinical trials of lung and breast cancers. Stomatitis is a common adverse event associated with Dato-DXd in these trials, and there are currently no formalized guidelines for its prevention and management. An expert consensus was needed to establish these guidelines.

**Methods:**

Fifteen experts participated in a modified three-round Delphi to achieve consensus on the prevention and management of Dato-DXd-related stomatitis in lung and breast cancer patients. Fourteen experts had managed Dato-DXd-induced stomatitis and served as investigators in the TROPION-Lung01, TROPION-Lung05, and TROPION-Breast01 phase III clinical trials. The first two rounds consisted of anonymized surveys with a mixture of open- and closed-ended questions, and the third round consisted of a consensus group meeting and 1:1 follow-up meetings.

**Findings:**

Experts recognized stomatitis as an important adverse event associated with several anticancer treatments and its impact on patients’ quality of life. Lung and breast cancer specialists noted similar manifestations of stomatitis with Dato-DXd, with breast cancer experts referencing similarities to everolimus-induced conditions and lung cancer experts reporting ulcerative lesions. There was consensus on the need for standardized guidelines to improve outcomes and reduce treatment disruptions. Agreed preventive measures emphasized patient education, oral hygiene, and prophylactic mouthwashes. Management strategies focused on patient-centered care, monitoring of symptoms, and timely interventions, such as the use of a steroid-containing mouthwash to maintain treatment schedules and quality of life.

**Interpretation:**

We used the expert consensus to provide guidelines for the prevention and management of stomatitis in patients treated with Dato-DXd.

**Putting research into context:**

**Evidence before this study:**

Dato-DXd has shown promising results in clinical trials for lung and breast cancers. However, many patients in these trials developed stomatitis, an adverse event that may impact quality of life and disrupt treatment regimens. The lack of standardized guidelines for dealing with stomatitis in this context necessitated the establishment of best practices via expert consensus.

**Added value of this study:**

This study provides peer-reviewed guidance for the prevention and management of stomatitis secondary to Dato-DXd. Most experts selected for this study had experience using this therapy and preventing and managing stomatitis associated with Dato-DXd and other anticancer treatments. Thus, the study has collected the perspectives of well-placed, expert clinicians and derived best practices pending the completion of clinical trials.

**Implications of all the available evidence:**

The development and implementation of this guidance and the education therein will lead to improved quality of life, greater treatment compliance, and better clinical outcomes.

**Supplementary Information:**

The online version contains supplementary material available at 10.1007/s00520-025-09805-y.

## Introduction

Oral mucositis/stomatitis (hereafter stomatitis) is an inflammation of the mucosal lining of any of the structures in the mouth and is characterized by ulceration, erythema, and impaired oral functioning, examples of which have been shown previously [1]. It is a common adverse event associated with many anticancer therapies, including chemotherapy, radiotherapy, immune checkpoint inhibitors, and targeted therapies like everolimus (67% incidence of all-grade stomatitis reported in patients with solid tumors) [1–6]. The presence of stomatitis may be superimposed with oral infections such as candidiasis and herpes simplex virus and clinical criteria for differential diagnosis has been established in guidelines [7, 8]. In severe cases, stomatitis negatively impacts patients’ quality of life, causing oral pain and inability to eat or drink ^9^. Dose delay or reduction, rescheduling, and cessation of treatment are often necessary consequences, and may impact cancer treatment dose density and outcomes [10].

Dato-DXd is an investigational humanized trophoblast cell-surface antigen 2 (TROP2)-directed monoclonal antibody–drug (topoisomerase I inhibitor) conjugate [11, 12]. It is being studied in several indications across solid tumors and has demonstrated promising clinical trial results in the lung and breast cancer settings. In the TROPION-Lung01 phase III trial (NCT04656652) [13], Dato-DXd improved median progression-free survival (mPFS) over docetaxel in patients with previously treated metastatic non-small cell lung cancer (mNSCLC) (4.4 vs 3.7 months). Similarly, Dato-DXd showed clinical benefit in mNSCLC patients with actionable genomic alterations (e.g., EGFRm) in the phase II TROPION-Lung05 clinical trial (NCT04484142) [14]. In the TROPION-Breast01 phase III trial (NCT05104866) [15], Dato-DXd demonstrated statistically significant improvement in mPFS (6.9 vs. 4.9 months) over the investigator’s choice of chemotherapy in patients with hormone receptor-positive/human epidermal growth factor receptor-2 negative metastatic breast cancer (HR + /HER2- mBC). Dato-DXd had a manageable safety profile in both trials with no new safety signals observed. The incidence rate of all-grade stomatitis was 55.2% in TROPION-Lung01 patients, 66% in TROPION-Lung05 patients, and 55.6% in TROPION-Breast01 patients. In TROPION-Breast01 patients, the median time to onset of stomatitis was 22 days and the median time to resolution was 36.5 days [16]. Grade ≥ 3 stomatitis was less common (6% and 7% in lung and breast cancer patients, respectively), and stomatitis resulted in a low rate (< 1%) of Dato-DXd discontinuation in both trials (Common Terminology Criteria for Adverse Events v5.0) [11, 12].

Toxicity management guidelines established for the TROPION trials to manage stomatitis secondary to Dato-DXd strongly recommended good oral hygiene practices and a steroid-containing mouthwash as prophylactic measures, and grade-dependent guides on dose reduction and treatment discontinuation [1]. The SWISH study, which examined the management of stomatitis secondary to everolimus in breast cancer patients, also offers some guidance for healthcare providers. In this phase II study, dexamethasone mouthwash (10 ml, 0·5 mg/5 ml solution 4 × /day, 2 min per time) was strongly recommended and reduced the incidence and severity of stomatitis over 8 weeks [17].

General guidelines are available for the prevention and management of stomatitis secondary to anticancer treatment and describe multimodal approaches with an emphasis on preventative measures broadly agreed upon [18]. These measures include patient education, good oral hygiene practices, and avoiding damage to the mucosa by avoiding ill-fitting prostheses and spicy, hard, or sharp food [19, 20]. However, these guidelines are not standardized, as they contain no consensus on the optimal approach for preventing and managing stomatitis [18], ultimately leaving treatment decisions to the expertise and experience of healthcare providers. They also lack the patient perspective in mucositis assessment and treatment decisions, which more focused guidelines could benefit from [21]. Overall, these guidelines have several limitations that cumulatively represent a significant knowledge gap.

For emerging therapies like Dato-DXd that have shown promising results in clinical trials [11, 12], collating and communicating the insights of the small pool of experienced healthcare providers is essential for patient care when the therapy becomes more widely available. The knowledge gap caused by the lack of formalized expert-led guidelines for the prevention and management of stomatitis secondary to Dato-DXd may impact clinician confidence in using this therapy and could lead to reduced quality of life and poorer patient outcomes due to dose reduction or treatment rescheduling.

This study aims to establish an expert consensus on best practices for the prevention, identification, diagnosis, and management of stomatitis secondary to Dato-DXd treatment in patients with cancer and to provide a set of best-practice guidelines based on these findings.

## Methods

### Overview of modified Delphi process

The Delphi consensus method is an iterative process of establishing a consensus among multidisciplinary experts on a specific topic through sequential surveys and structured interspersed feedback [22]. In each round, experts rate their agreement with a statement and can give feedback and revise their responses until a consensus is reached. The Delphi method has been employed to generate cancer treatment guidelines in many contexts [23–26]. In the case of stomatitis secondary to Dato-DXd treatment, the Delphi method brings the advantage of consensus-making to an area where there is limited empirical evidence but for which there is an unmet need.

The traditional Delphi method remains anonymous throughout. However, a modified version was performed with a panel discussion after the first two rounds of anonymized surveys. This approach provided deeper insights into the management of stomatitis that may have been lost in a fully anonymized survey-based method. Similar modifications have been implemented in the past [24]. The questionnaires were designed to be completed within one hour to minimize dropouts, thereby widening the scope of expert input.

### Expert panel selection

The quality of the output from the Delphi method is dependent on the depth and breadth of the available expertise. To this end, 15 experts were selected, including eight lung cancer specialists, six breast cancer specialists, and one oral medicine specialist. Fourteen experts served as investigators on the TROPION-Lung01, TROPION-Lung05, and/or TROPION-Breast01 trials and have managed Dato-DXd-induced stomatitis (Table 1).

**Table 1:** Expert panel characteristics

Characteristic	*n* (%)
**Gender**	
Man	7 (47)
Woman	8 (53)
**Primary field**	
Medical oncology	14 (93)
Oral medicine	1 (7)
**Subspecialty**	
Breast cancer	6 (40)
Lung cancer	8 (53)
Oral health specialist	1 (7)
**Years practicing**	
≤ 10	1 (7)
10–20	8 (53)
≥ 20	6 (40)
**Country**	
USA	14 (100)
**Region**	
Midwest	1 (7)
Northeast	6 (40)
South	5 (33)
West	3 (20)
**Practice setting**	
Academic	11 (73)
Community	2 (13)
Academic and community	1 (7)
Private and community	1 (7)
**Participation in the Dato-DXd TROPION clinical program**	
Yes	14 (93)
No	1 (7)

Other criteria for selection included:

- Advisership or membership in a national or international society focused on lung cancer, breast cancer, or oral medicine, with active contributions to guideline development within the past 5 years.
- Publication in a peer-reviewed journal on the treatment of patients with NSCLC, breast cancer, and/or stomatitis within the past 5 years.
- Actively treating and managing adverse events in lung or breast cancer patients.

Delphi rounds

Round one consisted of a 34-question survey, including questions further divided into sub questions. The questions were organized into nine subtopics with the following goals:

- Establishing the impact and presentation of stomatitis and alignment on the goals of future guidelines.
- Developing an understanding of best practices for the prevention, diagnosis, and treatment of stomatitis in patients receiving Dato-DXd.
- Exploring strategies for managing patients with stomatitis.

A combination of closed- and open-ended questions was used to gain a broader picture of the challenges of stomatitis while focusing on immediate areas of consensus. The anonymized results of round one were analyzed qualitatively to identify topics of consensus and areas to explore further in round two. Experts were provided with consolidated feedback from round one to allow them to understand and reflect on areas of agreement and disagreement.

Round two consisted of a 21-question survey, including questions further divided into sub-questions. A combination of closed- and open-ended questions were organized into four subtopics with the goal of achieving consensus on prophylactic measures, management approaches, and considerations for modifying the Dato-DXd regimen in the presence of stomatitis. Experts responded to closed-ended questions in rounds one and two using a nine-point Likert scale (described below). The anonymized results of round two were analyzed qualitatively to identify topics of consensus that were not carried forward for further discussion in the final round.

**Fig. 1:**
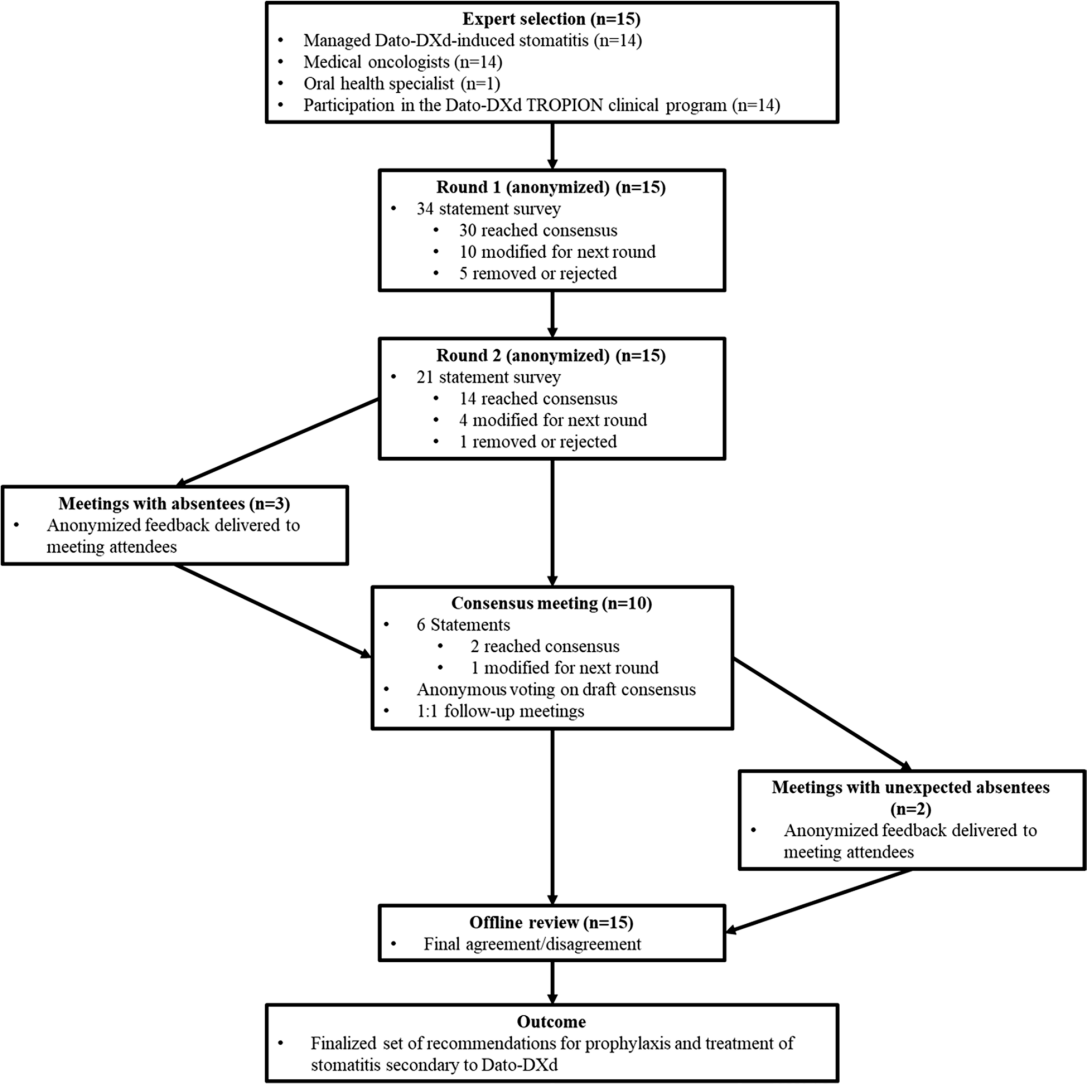
Flow of the modified Delphi process to reach consensus.

Round three consisted of a meeting and alignment sessions among the experts. Insights from round two were used to generate draft consensus statements, which were subsequently modified and voted upon anonymously during the consensus meeting. Statements where consensus was not reached were further discussed to determine the majority opinions. These consensus statements were distributed to all experts for offline review to document their final agreement or disagreement (Fig. 1).

### Likert scoring

For closed questions, experts rated their agreement/disagreement on a nine-point Likert scale, with a 1 indicating that they “strongly disagree with the statement” and a 9 representing that they “strongly agree with the statement”. For analysis, scores from 1 to 3 indicated *disagree*, 4–6 indicated *neither agree nor disagree*, and 7–9 indicated *agree*. Non-responses were also recorded. Consensus agreement was defined as ≥ 80% of experts reporting a score of ≥ 7, and consensus disagreement was defined as ≥ 80% of experts reporting a score of ≤ 3. Statements that scored outside these ranges at the end of the study were defined as instances where consensus could not be reached.

### Role of the funding source

The funding source played no role in the design or implementation of the study but only reviewed its final outcomes. A third party, Charles River Associates, was commissioned to design and oversee the study. This approach ensured that experts remained unbiased and were not influenced to align their responses with the sponsor’s interests.

## Results

### Summary of Delphi rounds

**Fig. 2:**
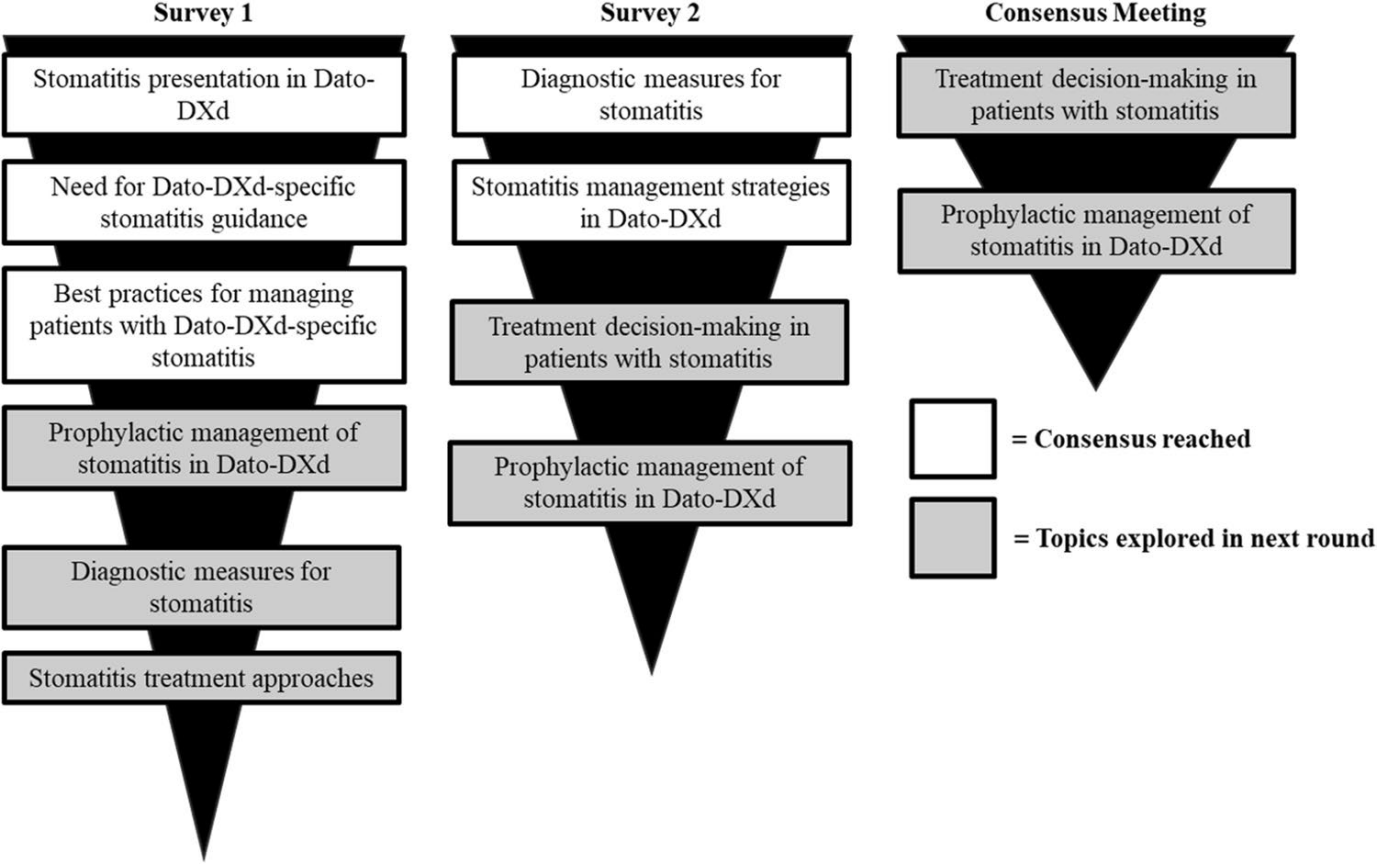
Flow of key statements across the rounds.

Thirty statements from round one reached consensus, and ten were modified/carried over to round two. Fourteen statements from round two achieved consensus, and four were discussed in the round three meeting, where consensus was reached on two (Tables 2, 3, 4, 5, and 6 and Supplementary Table 1). After the meeting, consensus was achieved on a further four revised statements. Individual meetings were conducted with three experts who could not attend the consensus meeting, and their perspectives were anonymously communicated to the ten participating experts. Subsequent meetings were held with two experts who were unexpectedly absent from the consensus meeting (Fig. 1). Figure 2 summarizes the flow of key statements across rounds, and Supplementary Table 2 outlines their evolution. Expert scores for each question are reported in Supplementary Tables 3 and 4. Summarized data for each question is reported in Supplementary Tables 5 and 6.

**Table 2:** Summary of statements relating to the knowledge gap for stomatitis management for which there was a consensus agreement across Delphi rounds

Key topic	Sub-question	% agree
**Round 1**		
I believe guidance on how to manage stomatitis in patients receiving Dato-DXd treatment would help healthcare professionals and the care team improve patients’ quality of life		93
I believe guidance on how to prevent and manage stomatitis in patients receiving Dato-DXd treatment would help improve clinical outcomes and mitigate dose reductions, treatment delays/interruptions, or treatment discontinuation		93
My goals when managing stomatitis in patients receiving Dato-DXd include:	Preventing or reducing incidence of stomatitis	100
	Managing pain associated with stomatitis	93
	Preventing complications associated with stomatitis	86
	Maintaining the patient’s oral function (i.e., ability to open mouth, speak, and swallow)	100
	Optimizing the patient’s quality of life	100
	Limiting dose reductions, delays, or treatment interruptions	86

**Table 3:** Summary of statements relating to stomatitis presentation in anticancer therapies and Dato-DXd for which there was a consensus agreement across Delphi rounds

Key topic	Sub-question	% agree
**Round 1**		
Stomatitis is a known adverse event associated with several oncology treatments for multiple cancer types, including NSCLC and breast cancer		93
In my experience, stomatitis can have a significant impact on patient quality of life and should be managed appropriately in patients receiving anticancer treatment		100
In my experience, complications associated with stomatitis can generally be mitigated when adhering to preventive, treatment, and management guidelines		87
In my experience, poorly managed stomatitis can impact patient clinical outcomes, including:	Malnourishment	100
	Weight loss	100
	Dysphagia	93
The onset of stomatitis associated with Dato-DXd treatment most commonly occurs within the initial treatment cycles of Dato-DXd, however, stomatitis can appear at any point throughout the treatment		93
Stomatitis associated with Dato-DXd treatment can be persistent and needs close management		93
I have patients who do not experience stomatitis when treated with Dato-DXd		86
**Round 2**		
[Breast Cancer Experts only] When determining my stomatitis prevention and treatment strategies for Dato-DXd, I would reference Everolimus guidance for best practices		100

**Table 4:** Summary of statements relating to diagnostic measures and monitoring for stomatitis for which there was a consensus agreement across Delphi rounds

Key topic	Sub-question	% agree
**Round 1**		
Upon Dato-DXd treatment initiation, I would encourage patients to perform regular self-checks to identify the onset of stomatitis and immediately report any signs or symptoms		93
I would use the following as primary methods of diagnosing stomatitis and staging during the Dato-DXd regimen	Mouth examination prior to each infusion	93
	Patient-reported symptoms	100
**Round 2**		
When determining if a patient has stomatitis, a physical examination and patient-reported symptoms are sufficient to make a diagnosis		100
For the following Dato-DXd patients, I would monitor for signs of stomatitis at the following frequency:	Mild presentation of stomatitis, 1 × per treatment cycle (3 weeks)	93
	Moderate presentation of stomatitis, 1 × per treatment cycle (3 weeks)	93
	Severe presentation of stomatitis, 1 × per treatment cycle (3 weeks)	93

**Table 5:** Summary of statements relating to preventative measures for stomatitis for which there was a consensus agreement across Delphi rounds

Key topic	Sub-question	% agree
**Round 1**		
I would educate my patients on the following issues prior to Dato-DXd treatment initiation:	Stomatitis awareness	100
	Oral care (e.g., brushing and flossing teeth, dietary changes)	93
	Preventive measures (e.g., mouthwashes)	100
	Early recognition & monitoring of symptoms	100
Before the first cycle of Dato-DXd treatment and continuing throughout the treatment, I would prescribe a set of preventive measures to reduce the likelihood and severity of stomatitis		93
As part of my stomatitis prevention strategy, I would recommend behavioral and additional prophylactic oral care to my patients prior to the first cycle and continuing throughout Dato-DXd treatment, including:	Brushing their teeth twice daily after meals and bedtime with a soft toothbrush	80
	Daily use of a prophylactic steroid-containing mouthwash (e.g., dexamethasone oral solution or a similar steroid)	87
I would continue preventive measures throughout the Dato-DXd treatment and on top of any treatment measures prescribed to manage stomatitis of any grade		93
**Round 2**		
As part of my prophylactic strategy to reduce the likelihood and severity of stomatitis, I would recommend “do no harm” behavioral changes for my patient, including:	Teeth brushing	92
	Rinsing their mouth with water	85
	Avoiding acidic or crunchy foods	85
	Cryotherapy (ice chips or ice water held in the patient’s mouth)	92
	Rinsing with a bicarbonate solution	85
I believe that only Dato-DXd patients with the following attributes should receive a prophylactic regimen to reduce the likelihood and severity of stomatitis	All patients starting Dato-DXd	100
As part of my prophylactic and ongoing management strategy of stomatitis, I would recommend a dexamethasone mouth rinse to my Dato-DXd patients		86
In the absence of a steroid mouth rinse, I would recommend a bland (i.e., non-alcoholic and/or bicarbonate-containing) mouth rinse to my Dato-DXd patients		86
**Round 3/post**		
I would recommend that my patients swish and spit a recommended steroid mouth rinse 3 to 4 × per day to prevent onset and manage stomatitis, starting on day 1 of treatment and throughout the course of treatment		··
I would recommend that my patients swish 1–2 min and then spit a recommended steroid mouth rinse		··

**Table 6:** Summary of statements relating to best practices for managing patients with stomatitis for which there was a consensus agreement across Delphi rounds

Key topic	Sub-question	% agree
**Round 1**		
Based on current guidelines for stomatitis management, I would consider the following treatments for mild stomatitis in patients receiving Dato-DXd treatment:	2% viscous lidocaine	87
	Steroid containing mouth rinse	93
	Magic mouthwash	93
Based on current guidelines for stomatitis management, I would consider the following treatments for moderate stomatitis in patients receiving Dato-DXd treatment in addition to treatments considered for mild stomatitis	Systemic opioids	93
	Magic mouthwash	93
Based on current guidelines for stomatitis management, I would consider the following treatments for severe stomatitis in patients receiving Dato-DXd treatment in addition to treatments considered for mild or moderate stomatitis:	Systemic opioids	92
For patients experiencing severe stomatitis while receiving Dato-DXd treatment, I would consider:	Delaying/interrupting Dato-DXd treatment	93
	Reducing Dato-DXd dose	86
I believe that educating care team members who spend significant time with the patients (e.g., nurses) on the prevention, identification, and management of stomatitis is important		100
I would discuss the patient’s stomatitis presentation and management strategies with the care team prior to stomatitis management initiation		93
When making decisions on the management of stomatitis or potential changes to the Dato-DXd treatment regimen, the patient’s quality of life should be a key consideration		100
Prior to each Dato-DXd infusion, I would ask the patient about their stomatitis prophylaxis / treatment adherence and quality of life to inform if other stomatitis interventions are needed		100
I would evaluate the following patient outcomes while managing patients experiencing stomatitis while receiving Dato-DXd treatment:	Pain level	100
	Weight	93
	Nutritional status	87
	Quality of life	100
Stomatitis management should be adapted to the individual patient due to variability in presentation and differing levels of pain tolerance		93
I would discuss prophylactic and management approaches for managing stomatitis with my patient to determine which approaches they are comfortable with and can access		93
Given adequate guidance on the management of stomatitis in patients receiving Dato-DXd treatment, I would consider the risk of stomatitis as a manageable adverse event		93
Given adequate guidance on the management of stomatitis in patients receiving Dato-DXd treatment, I would be comfortable prescribing Dato-DXd to my patients		93
Based on my clinical experience, I believe stomatitis seen in patients receiving Dato-DXd treatment can be adequately controlled with the preventive and treatment measures outlined in this study		86
**Round 2**		
I would recommend increasing the prophylactic and on-going stomatitis management strategies and NOT alter Dato-DXd treatment regimen upon seeing the following symptoms:	A patient reports mild oral pain, no ulceration, and limited* change in diet needed without any interference in oral intake	86
I would consider DELAYING Dato-DXd treatment regimen upon seeing the following symptoms:	A patient reports severe oral pain, ulceration, and significant interference with oral intake	92
	A patient exhibits signs of dehydration	83
	A patient experiences significant weight loss	83
	A patient has severe pain and is hospitalized for stabilization and/or pain control	82
I would consider REDUCING DOSAGE of Dato-DXd treatment regimen upon seeing the following symptoms:	A patient experiences significant weight loss	89
	A patient has severe pain and is hospitalized for stabilization and/or pain control	82
I would consider DISCONTINUATION of the Dato-DXd treatment regimen upon seeing the following symptoms:	A patient has severe pain and is hospitalized for stabilization and/or pain control	86
**Round 3/post**		
If Grade 1 stomatitis occurs, I would continue the steroid mouth rinse, consider using a steroid dental gel and suggest avoiding crunchy/spicy foods		··
With Grade 2 OM/S with prophylaxis adherence, I would hold Dato-DXd until symptomatic improvement, and then reinitiate with a dose reduction with the steroid mouth-rinse prophylaxis With Grade 2 OM/S without prophylaxis adherence, I would hold Dato-Dxd, initiate with a steroid-containing mouth rinse and upon symptomatic improvement, resume Dato-DXd at the same dose with the steroid mouth-rinse prophylaxis With Grade 3 OM/S, I would hold Dato-DXd until symptomatic resolution, then consider reinitiate with a dose reduction with the steroid mouth-rinse prophylaxis		··

### Knowledge gap for stomatitis management

Effective treatments for lung and breast cancer are lacking, especially for refractory or late-stage disease. Where treatments are available, improving outcomes and maintaining efficacy are critical for increasing patient survival. While Dato-DXd demonstrated potential improvement of efficacy, experts agreed that the lack of standardized guidelines for preventing and managing stomatitis secondary to Dato-DXd constituted an unmet clinical need and a barrier to optimal patient care. They further agreed that establishing guidelines would improve clinical outcomes and prevent the need for dose reductions, rescheduling, and cessation of treatment associated with stomatitis [9].

The experts stated the primary goals were prevention and management of stomatitis, improved quality of life, and maintenance of treatment schedules, which align directly with the goal of this study. Statements relating to knowledge gap for stomatitis management for which there was a consensus agreement across Delphi rounds are summarized in Table 2.

### Stomatitis presentation in anticancer therapies and Dato-DXd

Experts broadly agreed with the current understanding of stomatitis as a common but non-ubiquitous adverse event of many anticancer treatments that can have significant implications on quality of life and treatment efficacy [3, 9]. Breast cancer experts demonstrated more familiarity and experience in managing stomatitis compared to lung cancer experts, reporting presentations similar to those associated with everolimus. It is important to note, however, that everolimus and Dato-DXd likely cause stomatitis through distinct pathophysiological mechanisms. Everolimus is an mTOR inhibitor, whereas Dato-DXd contains a topoisomerase I inhibitor. Both drug classes have been shown to induce an upregulation of inflammatory markers. However, the underlying mechanisms through which they cause stomatitis are multifactorial and not fully understood [27, 28]. Statements relating to stomatitis presentation for which there was a consensus agreement across Delphi rounds are summarized in Table 3.

### Diagnostic measures and monitoring for stomatitis

Experts highlighted that patients often reported stomatitis symptoms before manifesting visible symptoms. This helps to illustrate the importance of a patient-centered approach to diagnosis and monitoring of this adverse event. Oral inspection was reported as the primary mode of examination to be performed prior to each infusion and upon patient reported symptoms. Consensus was not reached that consultation with an oral health specialist was required for diagnosis except in cases of uncertainty. Instead, experts reached consensus that a physical examination and patient-reported symptoms are sufficient for diagnosis of stomatitis. Experts agreed on encouraging patients to perform regular self-checks for stomatitis and immediately report any signs or symptoms to their healthcare provider. Statements relating to diagnosis and monitoring for which there was a consensus agreement across Delphi rounds are summarized in Table 4.

### Preventive measures for stomatitis

Experts reached a consensus that patient education is essential for preventing stomatitis. They emphasized the importance of discussing key topics with patients before initiating Dato-DXd treatment, including the following:

- General awareness of stomatitis
- Routine oral care (i.e., teeth brushing and rinsing with water)
- Early symptom recognition and monitoring

Experts recommend regular check-ins with the care team, including follow-up calls by nurses, to ensure adherence to prophylactic measures and address early symptoms. They agreed that a prophylactic regimen should start on day 1 of the first Dato-DXd cycle and continue throughout treatment. Emphasis was placed on patient oral hygiene, including brushing teeth and using a prophylactic mouthwash. They agreed that oral cryotherapy can be considered a preventive strategy as described in other studies [19]. Consensus supported the use of a steroid-containing mouthwash, preferably with dexamethasone, or an inert bland alcohol-free wash, or one with sodium bicarbonate (Table 7). Statements relating to prevention for which there was a consensus agreement across Delphi rounds are summarized in Table 5.

**Table 7:** Suggested prophylactic regimen for stomatitis in Dato-DXd. The suggested regimen starts on day 1 of cycle 1 and continues for the duration of Dato-DXd treatment regimen.

Prophylaxis	Strongly suggested	Steroid-containing mouth rinse (e.g., Dexamethasone): Swish and spit 3–4 × per day for 1–2 min
		Oral hygiene: Teeth brushing, flossing, and rinsing with water
		Patient education: Stomatitis awareness, early signs and symptoms, and oral care routine
	May be considered	Cryotherapy: Ice chips, ice water, or popsicles held in the mouth for a few minutes before infusion, during the infusion, and for some time after the infusion
		Inert, bland rinses (e.g., alcohol-free bicarbonate): Swish and spit 3–4 × per day for 1–2 min prior to steroid-containing mouth rinse or instead of steroid-containing mouth rinse if unavailable

A change in diet (e.g., removing spicy/crunchy food) was not recommended unless oral sensitivity or ulceration occurs. The experts did not reach a consensus on the need for comprehensive professional oral examination at treatment initiation or the use of flossing as part of a “do no harm” prophylaxis regimen.

### Best practices for managing patients with stomatitis

With appropriate guidance, experts believe stomatitis is a manageable adverse event associated with Dato-DXd treatment. Experts agreed that decision-making should be patient-centered, considering stomatitis severity, impact on quality of life, and the potential risk of losing the efficacy of therapeutic intervention. The experts considered factors, such as the patient’s ability to eat and drink, improvement of stomatitis, symptom response, and overall well-being to determine if other interventions are necessary and to assess the potential impact of treatment delays. Importantly, experts agreed to measure the effects of stomatitis on patients treated with Dato-DXd by monitoring oral pain levels, weight loss, and nutritional status to ensure timely interventions.

Recommendations for stomatitis associated with Dato-DXd based on Common Terminology Criteria for Adverse Events (CTCAE) grading are outlined in Table 8. For grade 1 stomatitis (CTCAE), experts suggested diet modifications to avoid spicy, acidic, and crunchy foods, along with topical steroid-containing gels and steroid-containing mouthwashes. For grade 2 stomatitis (CTCAE) with prophylaxis adherence, they suggested delaying Dato-DXd treatment until symptoms improve, restarting at a reduced dosage, and using a steroid-containing mouthwash daily. This diverges slightly from the TROPION-Lung01 and TROPION-Breast01 trial guidelines, where grade 2 did not mandate a dose reduction but instead recommended that the clinician consider it. The same treatment is advised for grade 3 stomatitis (CTCAE), regardless of prophylaxis adherence. For grade 2 without prophylaxis adherence, they suggested delaying treatment until symptoms improve and use a steroid-containing mouthwash daily (Table 8). Statements relating to mucositis/stomatitis management for which there was a consensus agreement across Delphi rounds are summarized in Table 6.

**Table 8:** Suggested Stomatitis Management in Dato-DXd*. *Treatment approaches should be provided concurrently.

Suggested treatment	CTCAE Grade 1; asymptomatic or mild symptoms; intervention not indicated	With or without prophylaxis adherence	Diet modification: Avoidance of spicy, acidic, and crunchy foods
			Topical steroid gel: Used for spot treatment directly on mouth ulcers
			Steroid-containing mouth rinse: 3–4 × per day for 1–2 min
	CTCAE Grade 2; moderate pain or ulcer; not interfering with oral intake; moderate diet indicated	With prophylaxis adherence	Dato-DXd treatment delay: Until symptomatic improvement
			Reinitiate Dato-DXd at a reduced dose: Typically from 6 to 4 mg/kg
			Steroid-containing mouth rinse: 3–4 × per day for 1–2 min
		Without prophylaxis adherence	Dato-DXd treatment delay: Until symptomatic improvement
			Steroid-containing mouth rinse: 3–4 × per day for 1–2 min
	CTCAE Grade 3; severe pain, interfering with oral intake	With or without prophylaxis adherence	Dato-DXd treatment delay: Until symptomatic improvement
			Consider reinitiating Dato-DXd at a reduced dose: Typically from 6 to 4 mg/kg
			Steroid-containing mouth rinse: 3–4 × per day for 1–2 min
	CTCAE Grade 4; Life-threatening consequences; urgent intervention indicated		Not evaluated further as a grade 3 suggestion already indicates treatment delay

## Discussion

There is lack of published consensus on specific treatments for prevention and management of stomatitis, a common adverse event secondary to anticancer treatment that can impact patient outcomes. Efforts to generate empirical evidence on the optimal treatments for stomatitis secondary to Dato-DXd are underway. Without this evidence, expert opinions on Dato-DXd use and stomatitis management are vital for establishing best practices and improving patient outcomes, as the absence of consensus guidelines poses significant risks to treatment efficacy and quality of life.

The present study utilized a modified Delphi method to reach expert consensus on preventing and managing stomatitis associated with Dato-DXd in lung and breast cancer patients. The broad consensus achieved by experts with experience using Dato-DXd in this study enabled the development of best-practice recommendations for this adverse event, filling a significant gap in existing guidelines. This study is a vital contribution to the improvement of patient outcomes by reducing treatment dosage and scheduling changes while enhancing patient quality of life.

The expert consensus agreed with existing opinions on the importance of preventing and managing stomatitis. The experts emphasized the role of patients and caregivers in symptom control and adherence to treatment, using a holistic clinical management strategy. Patient centricity is fundamental to achieving improved outcomes, and digital tools that enable tracking and reporting of symptoms and ongoing education are promising solutions for enhancing patient adherence and achieving better healthcare outcomes [29].

The experts frequently cited the SWISH study as helping to guide their approach to managing and preventing stomatitis in the Dato-DXd trials. The study investigated the use of dexamethasone mouthwash to prevent everolimus-related stomatitis in women with hormone receptor-positive, HER2-negative metastatic breast cancer [17]. In the study, dexamethasone mouthwash (10 ml, 0·5 mg/5 ml solution 4 × /day, 2 min per time) substantially reduced the incidence and severity of everolimus-related stomatitis.

There is currently no Dato-DXd-specific data regarding the use of dexamethasone or steroid-containing mouthwashes. One expert expressed hesitation in recommending a steroid-containing mouthwash without clinical data. Despite similar ulcerative presentation, everolimus and Dato-DXd likely trigger stomatitis through distinct pathophysiologic mechanisms [27, 28]. Experts who have more experience with Dato-DXd acknowledged this data gap during the consensus meeting. Despite this, they agreed that the benefits of dexamethasone/steroid-containing mouthwashes outweigh the risks for patients receiving Dato-DXd. Thus, a consensus was reached to strongly suggest that patients use a steroid-containing mouthwash by swishing and spitting three to four times daily to prevent and manage stomatitis, with dexamethasone being the preferred corticosteroid. Prospective empirical evidence is needed to confirm clinical management strategies, and the optimal dosing regimen of dexamethasone mouthwash for Dato-DXd patients is being evaluated.

Some experts reported success with using “magic mouthwash” to treat patients. These mouthwashes contain different combinations of steroids, antihistamines, analgesics, antibiotics, and antifungals [30]. However, “magic mouthwashes” vary between institutions, and their formulations are not standardized. This makes it challenging to derive clinically useful information presenting a barrier to recommending “magic mouthwashes” in formal guidelines.

Consensus was achieved amongst experts for the use of steroid-containing mouth rinses. Despite not being currently included in guidelines for the treatment of oral mucositis secondary to cancer therapy, steroid-containing mouth rinses have been proven effective and safe in patients with oral mucositis on other cancer therapies [18]. Future and/or ongoing studies are expected to better inform this practice moving forward.

While some cancer management guidelines recommend oral cryotherapy, such as ice chips or popsicles, experts have mixed opinions about its use. During the consensus meeting, experts agreed that oral cryotherapy can be included in a “do no harm” prevention strategy and should be considered by interested practitioners. Those with experience using cryotherapy suggest initiating it a few minutes before infusion and continuing throughout the infusion period.

Regarding prophylaxis, consensus was not reached on the use of measures like zinc supplements, oral glutamine, honey, palifermin, and antibiotics/antifungals, despite being recommended in other guidelines [18, 31] . Similarly, consensus was not reached for the use of bacterial, fungal, or viral culture to diagnose stomatitis, despite other guidelines recommending these tests to identify causal pathogens of persistent or recurrent symptoms [32]. Experts deemed oral examination and patient-reported symptoms sufficient for diagnosis.

Our modified Delphi study also presented some limitations. The experts included in this study were from different specialty backgrounds, namely breast cancer and lung cancer, with one oral care specialist. The extent of experts’ training in oral care as well as their experience may therefore have impacted responses, potentially causing bias. The small expert pool also necessitated the selection of US-based experts only, increasing the chances of participants recognizing colleagues’ opinions or writing style, potentially causing bias. This bias could have been amplified by removing anonymity in the consensus meeting. However, flexibility was required to reach consensus on complex issues. Moreover, most statements reached consensus during the initial anonymized rounds and the study was performed by an independent third-party, not the sponsor.

Another limitation of this study was the absence of patient perspectives, resulting in a lack of insight into their experiences and preferences during treatment. Future research should focus on addressing patients’ concerns and needs, particularly regarding the management of adverse events.

In conclusion, experts agreed that stomatitis in patients receiving Dato-DXd treatment can be adequately controlled with the preventive and treatment measures outlined in this study. Thus, an important clinical need has been addressed using the best available evidence with insights from the experts experienced in managing Dato-DXd-associated stomatitis. More research is needed to generate evidence and effectively educate healthcare providers related to stomatitis management to further optimize clinical outcomes associated with Dato-DXd in lung and breast cancer patients.

## Supplementary Information

Below is the link to the electronic supplementary material.

ESM 1 (133 KB DOCX)

## Data Availability

No datasets were generated or analysed during the current study.
