# Supplementary material for: US expert Delphi consensus on the prevention and management of stomatitis in patients treated with datopotamab deruxtecan
Source: Support Care Cancer. 2025 Aug 5;33(9):756. doi: 10.1007/s00520-025-09805-y (PMC12325563; doi:10.1007/s00520-025-09805-y)
Supplement: Supplementary file 1 — (133 KB DOCX) [file 520_2025_9805_MOESM1_ESM.docx]

**Supplementary Material:**

**Table of Contents**

[Supplementary Table 1 Summary of statements for which there was a consensus disagreement across Delphi rounds 1](#_Toc175659698)

[Supplementary Table 2 Evolution of stomatitis management statements 2](#_Toc175659699)

[Supplementary Table 3 Results from the first survey, full Likert breakdown 13](#_Toc175659700)

[Supplementary Table 4 Results from the second survey, full Likert breakdown 19](#_Toc175659701)

[Supplementary Table 5 Results from the first survey, summary of scoring 24](#_Toc175659702)

[Supplementary Table 6 Results from the second survey, summary of scoring 29](#_Toc175659703)

[References 33](#_Toc175659704)

##

## Supplementary Table 1 Summary of statements for which there was a consensus disagreement across Delphi rounds

| **Key Topic** | **Sub-question** | **% disagree** |
| --- | --- | --- |
| **Round 1** | | |
| For patients experiencing severe stomatitis while receiving Dato-DXd treatment, I would consider: | Keeping the Dato-DXd treatment regimen as is | 100 |
| **Round 2** | | |
| I believe that only Dato-DXd patients with the following attributes should receive a prophylactic regimen to reduce the likelihood and severity of stomatitis: | Patients with a prior history of mouth sores | 86 |
|  | Patients aged 65+ years old | 86 |
|  | Patients with poor nutritional status | 86 |
|  | Patients with poor oral hygiene or other oral conditions | 86 |
|  | Patients with a prior history of smoking | 93 |
| For patients I would place on a prophylactic regimen, I would begin prophylaxis: | After Dato-DXd treatment initiation, in patients WITHOUT stomatitis symptoms | 100 |
|  | After Dato-DXd treatment initiation, in patients WITH stomatitis symptoms | 86 |
| I would recommend my Dato-DXd patients use the dexamethasone mouth rinse with the following frequency: | 1x per day | 100 |
|  | I would not recommend a dexamethasone mouth rinse to my Dato-DXd patients | 93 |
| I would recommend my Dato-DXd patients swish the dexamethasone mouth rinse in their mouth for the following period of time before discarding: | Under 1 minute | 86 |
|  | 2-3 minutes | 86 |
|  | I would not recommend a dexamethasone mouth rinse to my Dato-DXd patients | 93 |
| For the following Dato-DXd patients, I would monitor for signs of stomatitis at the following frequency: | Non-symptomatic - 1x per every 2 treatment cycles (6 weeks) | 86 |
|  | Non-symptomatic - Every 6 months | 100 |
|  | Non-symptomatic - Once a year | 100 |
|  | Non-symptomatic - I would only monitor once symptoms arise | 86 |
|  | Mild presentation of stomatitis - 1x per every 2 treatment cycles (6 weeks) | 93 |
|  | Mild presentation of stomatitis - Every 6 months | 100 |
|  | Mild presentation of stomatitis - Once a year | 100 |
|  | Mild presentation of stomatitis - I would only monitor once symptoms arise | 100 |
|  | Moderate presentation of stomatitis - 1x per every 2 treatment cycles (6 weeks) | 93 |
|  | Moderate presentation of stomatitis - Every 6 months | 100 |
|  | Moderate presentation of stomatitis - Once a year | 100 |
|  | Moderate presentation of stomatitis - I would only monitor once symptoms arise | 100 |
|  | Severe presentation of stomatitis - 1x per every 2 treatment cycles (6 weeks) | 93 |
|  | Severe presentation of stomatitis - Every 6 months | 100 |
|  | Severe presentation of stomatitis - Once a year | 100 |
|  | Severe presentation of stomatitis - I would only monitor once symptoms arise | 100 |
| I would consider REDUCING DOSAGE of Dato-DXd treatment regimen upon seeing the following symptoms: | A patient reports mild oral pain, no ulceration, and limited* change in diet needed without any interference in oral intake*Includes avoiding spicy or acidic food | 86 |
| I would consider DISCONTINUATION of the Dato-DXd treatment regimen upon seeing the following symptoms: | A patient reports moderate oral pain, ulceration, and a moderate change in diet needed without any interference in oral intake | 92 |

## Supplementary Table 2 Evolution of stomatitis management statements

**Key:**

|  | **Consensus Reached** (≥80% of experts agreed) |
| --- | --- |
|  | **Consensus Reached Against** (≥80% of experts disagreed) |
|  | **Consensus Not Reached** (20% - 79% experts agreed) |
|  | **No Further Statements Created** |

| **Survey 1** | **Survey 2** | **Consensus meeting** | **Modified Final statement from consensus meeting** |
| --- | --- | --- | --- |
| **OM/S Presentation in Dato-DXd – Across anticancer-therapies** | | | |
| Stomatitis is a known adverse event associated with several oncology treatments for multiple cancer types, including NSCLC and breast cancer |  |  |  |
| In my experience, stomatitis can have a significant impact on patient quality of life and should be managed appropriately in patients receiving anticancer treatment |  |  |  |
| In my experience, complications associated with stomatitis can generally be mitigated when adhering to preventive, treatment, and management guidelines |  |  |  |
| In my experience, poorly managed stomatitis can impact patient clinical outcomes, including: malnourishment, weight loss, dysphagia, oral candidiasis and herpes simplex virus (HSV) |  |  |  |
| **OM/S Presentation in Dato-DXd – Dato-DXd specific** | | | |
| The onset of stomatitis associated with Dato-DXd treatment most commonly occurs within the initial treatment cycles of Dato-DXd, however, stomatitis can appear at any point throughout the treatment |  |  |  |
| Stomatitis associated with Dato-DXd treatment can be persistent and needs close management |  |  |  |
| I have patients who do not experience stomatitis when treated with Dato-DXd |  |  |  |
| **Prevention measures for OM/S – Patient education** | | | |
| I would educate my patients on the following issues prior to Dato-DXd treatment initiation: stomatitis awareness, oral care (e.g., brushing and flossing teeth, dietary changes), preventive measures (e.g., mouthwashes), and early recognition & monitoring of symptoms | I believe that only Dato-DXd patients with the following attributes should receive a prophylactic regimen to reduce the likelihood and severity of stomatitis: all patients starting Dato-DXd |  |  |
|  | I believe that only Dato-DXd patients with the following attributes should receive a prophylactic regimen to reduce the likelihood and severity of stomatitis: patients with a prior history of mouth sores, patients with currently active mouth sores, patients aged 65+ years old, patients with poor nutritional status, patients with poor oral hygiene or other oral conditions, and patients with a prior history of smoking |  |  |
| **Prevention measures for OM/S – Timing of prevention measures** | | | |
| Before the first cycle of Dato-DXd treatment and continuing throughout the treatment, I would prescribe a set of preventive measures to reduce the likelihood and severity of stomatitis | For patients I would place on a prophylactic regimen, I would begin prophylaxis: Prior to Dato-DXd treatment initiation and at Dato-DXd treatment initiation | I would start the prophylaxis regimen at day 1 of cycle 1 of Dato-DXd treatment |  |
|  | For patients I would place on a prophylactic regimen, I would begin prophylaxis: Prior to Dato-DXd in patients WITHOUT stomatitis symptoms, or after Dato-DXd treatment initiation, in patients WITH stomatitis symptoms |  |  |
| I would continue preventive measures throughout the Dato-DXd treatment and on top of any treatment measures prescribed to manage stomatitis of any grade |  |  |  |
| **Prevention measures for OM/S – Behavioral and prophylactic prevention measures** | | | |
| As part of my stomatitis prevention strategy, I would recommend behavioral and additional prophylactic oral care to my patients prior to the first cycle and continuing throughout Dato-DXd treatment, including: Brushing their teeth twice daily after meals and bedtime with a soft toothbrush and daily use of a prophylactic steroid-containing mouthwash (e.g., dexamethasone oral solution or a similar steroid) | As part of my prophylactic and ongoing management strategy of stomatitis, I would recommend a dexamethasone mouth rinse to my Dato-DXd patients |  |  |
|  | I would recommend my Dato-DXd patients use the dexamethasone mouth rinse with the following frequency: 2x per day, 3x per day, or 4x per day | I would recommend that my patients swish and spit a recommended steroid mouth rinse 4x per day to prevent onset of stomatitis | I would recommend that my patients swish and spit a recommended steroid mouth rinse 3 to 4x per day to prevent onset and manage stomatitis, starting on day 1 of treatment and throughout the course of treatment |
|  | I would recommend my Dato-DXd patients use the dexamethasone mouth rinse with the following frequency: 1x per day or I would not recommend a dexamethasone mouth rinse to my Dato-DXd patients |  |  |
|  | I would recommend my Dato-DXd patients swish the dexamethasone mouth rinse in their mouth for the following period of time before discarding: 1 minute, 1-2 minutes | I would recommend that my patients swish for 1-2 minutes and then spit a recommended steroid mouth rinse |  |
|  | I would recommend my Dato-DXd patients swish the dexamethasone mouth rinse in their mouth for the following period of time before discarding: Under 1 minute, 2-3 minutes, or I would not recommend a dexamethasone mouth rinse to my Dato-DXd patients |  |  |
|  | I would recommend the following dexamethasone mouth rinse to my Dato-DXd patients: Commercially available or All of the above, no preference |  |  |
|  | I would recommend the following dexamethasone mouth rinse to my Dato-DXd patients: Compounded in a hospital pharmacy or compounded in a local pharmacy |  |  |
|  | If dexamethasone rinse is not available, I would recommend another steroid mouth rinse which does not contain alcohol to my Dato-DXd patients. |  |  |
| As part of my stomatitis prevention strategy, I would recommend behavioral and additional prophylactic oral care to my patients prior to the first cycle and continuing throughout Dato-DXd treatment, including: Flossing their teeth once daily, rinsing their mouth with tap water, daily rinse with a saline solution, daily rinse with a sodium bicarbonate solution, daily use of a prophylactic non-steroidal anti-inflammatory mouthwash (e.g., benzydamine), and prophylactic cryotherapy (ice chips or ice water held in the patient’s mouth throughout the infusion) | As part of my prophylactic strategy to reduce the likelihood and severity of stomatitis, I would recommend ‘do no harm’ behavioral changes for my patient, including: Teeth brushing, rinsing their mouth with water, avoiding acidic or crunchy foods, cryotherapy (ice chips or ice water held in the patient’s mouth), rinsing with a bicarbonate solution |  |  |
|  | As part of my prophylactic strategy to reduce the likelihood and severity of stomatitis, I would recommend ‘do no harm’ behavioral changes for my patient, including: flossing |  |  |
|  | In the absence of a steroid mouth rinse, I would recommend a bland (i.e., non-alcoholic and/or bicarbonate-containing) mouth rinse to my Dato-DXd patients. |  |  |
| As part of my stomatitis prevention strategy, I would recommend behavioral and additional prophylactic oral care to my patients prior to the first cycle and continuing throughout Dato-DXd treatment, including: zinc supplements, oral glutamine, honey, palifermin (recombinant human keratinocyte treatment), and antibiotics and antifungals |  |  |  |
| **Diagnostic measures for OM/S – Diagnosis methods** | | | |
| Upon Dato-DXd treatment initiation, I would encourage patients to perform regular self-checks to identify the onset of stomatitis and immediately report any signs or symptoms | For Dato-DXd patients with, mild, moderate, and severe stomatitis, I would monitor for signs of stomatitis at the following frequency: 1x per treatment cycle. |  |  |
|  | For Dato-DXd patients with non-symptomatic, mild, moderate, and severe stomatitis, I would monitor for signs of stomatitis at the following frequency: 1x per every 2 treatment cycles, every 6 months, once a year, I would only monitor once symptoms arise |  |  |
|  | For Dato-DXd patients with non-symptomatic stomatitis, I would monitor for signs of stomatitis at the following frequency: 1x per treatment cycle |  |  |
| I would use the following as primary methods of diagnosing stomatitis and staging during the Dato-DXd regimen: mouth examination prior to each infusion and patient-reported symptoms |  |  |  |
| I would use the following as primary methods of diagnosing stomatitis and staging during the Dato-DXd regimen: bacterial, fungal, or viral cultures |  |  |  |
| I would recommend a comprehensive professional oral examination by an oral health professional at Dato-DXd treatment initiation. | When determining if a patient has stomatitis, a physical examination and patient-reported symptoms are sufficient to make a diagnosis. |  |  |
| **Diagnostic measures for OM/S – Need for additional consultation** | | | |
| Upon the first signs of stomatitis, I would consult with an oral health specialist to confirm the diagnosis |  |  |  |
| **Treatment measures for OM/S – Mild presentation** | | | |
| Based on current guidelines for stomatitis management, I would consider the following treatments for mild stomatitis in patients receiving Dato-DXd treatment: 2% viscous lidocaine, magic mouthwash, and steroid containing mouth rinse |  |  |  |
| Based on current guidelines for stomatitis management, I would consider the following treatments for mild stomatitis in patients receiving Dato-DXd treatment: Cryotherapy, chlorhexidine, topical NSAIDs, topical corticosteroids, sugarless chewing, salivary substitutes, or sialagogues |  |  |  |
|  |  | If stomatitis occurs, I would increase the frequency of the recommended steroid mouth rinse to every other hour | If Grade 1 stomatitis occurs, I would continue with the steroid mouth rinse, consider using a steroid dental gel and suggest avoiding crunchy/spicy foods |
| I would not reduce the Dato-DXd dose or interrupt the treatment regimen for my patients receiving Dato-DXd treatment who present with a mild clinical presentation of stomatitis | I would recommend increasing the prophylactic and on-going stomatitis management strategies and NOT alter Dato-DXd treatment regimen upon seeing the following symptoms: A patient reports mild oral pain, no ulceration, and limited change in diet (includes avoiding spicy foods) needed without any interference in oral intake |  |  |
|  | I would recommend increasing the prophylactic and on-going stomatitis management strategies and NOT alter Dato-DXd treatment regimen upon seeing the following symptoms: A patient reports mild oral pain, slight ulceration, and limited* change in diet needed without any interference in oral intake; A patient reports mild oral pain, slight ulceration, and some* change in diet needed without any interference in oral intake; A patient reports moderate oral pain, ulceration, and a moderate change in diet needed without any interference in oral intake; A patient reports severe oral pain, ulceration, and significant interference with oral intake |  |  |
| **Treatment measures for OM/S – Moderate presentation** | | | |
| Based on current guidelines for stomatitis management, I would consider the following treatments for moderate stomatitis in patients receiving Dato-DXd treatment in addition to treatments considered for mild stomatitis: systemic opioids and magic mouthwash |  |  |  |
| Based on current guidelines for stomatitis management, I would consider the following treatments for moderate stomatitis in patients receiving Dato-DXd treatment in addition to treatments considered for mild stomatitis: mucosal coating agents, antibiotics, or antifungals, intralesional steroid injections, topically applied aloe |  |  |  |
| For patients experiencing moderate stomatitis while receiving Dato-DXd treatment, I would consider: Delaying/interrupting Dato-DXd treatment, reducing Dato-DXd dose, keeping the Dato-DXd treatment regimen as is or discontinuing Dato-DXd treatment | I would consider DELAYING Dato-DXd treatment regimen upon seeing the following symptoms: A patient reports severe oral pain, ulceration, and significant interference with oral intake; A patient exhibits signs of dehydration; A patient experiences significant weight loss; A patient has severe pain and is hospitalized for stabilization and/or pain control |  |  |
|  | I would consider DELAYING Dato-DXd treatment regimen upon seeing the following symptoms: A patient reports mild oral pain, no ulceration, and limited* change in diet needed without any interference in oral intake*Includes avoiding spicy or acidic food; A patient reports mild oral pain, slight ulceration, and some* change in diet needed without any interference in oral intake; c. A patient reports mild oral pain, slight ulceration, and some* change in diet needed without any interference in oral intake*Some solid foods may be limited and some change to diet may be required ; A patient reports moderate oral pain, ulceration, and a moderate change in diet needed without any interference in oral intake | I would delay or consider reducing the Dato-DXd dose in patients with moderate (Grade 2) or severe (Grade 3) stomatitis | With Grade 2 OM/S with prophylaxis adherence, I would hold Dato-DXd until symptomatic improvement, and then reinitiate with a dose reduction with the steroid mouth-rinse prophylaxis ​ |
|  |  |  | With Grade 2 OM/S without prophylaxis adherence, I would hold Dato-DXd, initiate with a steroid-containing mouth rinse and upon symptomatic improvement, resume Dato-DXd at the same dose with the steroid mouth-rinse prophylaxis |
|  | I would consider REDUCING DOSAGE of Dato-DXd treatment regimen upon seeing the following symptoms: A patient experiences significant weight loss; A patient has severe pain and is hospitalized for stabilization and/or pain control |  |  |
|  | I would consider REDUCING DOSAGE of Dato-DXd treatment regimen upon seeing the following symptoms: A patient reports mild oral pain, slight ulceration, and limited* change in diet needed without any interference in oral intake*Includes avoiding spicy or acidic food; A patient reports mild oral pain, slight ulceration, and some* change in diet needed without any interference in oral intake*Some solid foods may be limited and some change to diet may be required; A patient reports moderate oral pain, ulceration, and a moderate change in diet needed without any interference in oral intake; A patient reports severe oral pain, ulceration, and significant interference with oral intake |  |  |
|  | I would consider REDUCING DOSAGE of Dato-DXd treatment regimen upon seeing the following symptoms: A patient reports mild oral pain, no ulceration, and limited* change in diet needed without any interference in oral intake*Includes avoiding spicy or acidic food |  |  |
| **Treatment measures for OM/S – Severe presentation** | | | |
| Based on current guidelines for stomatitis management, I would consider the following treatments for severe stomatitis in patients receiving Dato-DXd treatment in addition to treatments considered for mild or moderate stomatitis: systemic oral corticosteroids, daily low-level laser therapy |  |  |  |
| Based on current guidelines for stomatitis management, I would consider the following treatments for severe stomatitis in patients receiving Dato-DXd treatment in addition to treatments considered for mild or moderate stomatitis: Systemic opioids |  |  |  |
| For patients experiencing severe stomatitis while receiving Dato-DXd treatment, I would consider: Delaying/interrupting Dato-DXd treatment or Reducing Dato-DXd dose |  |  |  |
| For patients experiencing severe stomatitis while receiving Dato-DXd treatment, I would consider: Discontinuing Dato-DXd treatment |  | I would delay or consider reducing the Dato-DXd dose in patients with moderate (Grade 2) or severe (Grade 3) stomatitis | With Grade 3 OM/S, I would hold Dato-DXd until symptomatic resolution, then consider reinitiating with a dose reduction with the steroid mouth-rinse prophylaxis |
| For patients experiencing severe stomatitis while receiving Dato-DXd treatment, I would consider: Keeping the Dato-DXd treatment regimen as is | I would consider DISCONTINUATION of the Dato-DXd treatment regimen upon seeing the following symptoms: A patient has severe pain and is hospitalized for stabilization and/or pain control |  |  |
|  | I would consider DISCONTINUATION of the Dato-DXd treatment regimen upon seeing the following symptoms: A patient reports severe oral pain, ulceration, and significant interference with oral intake; A patient exhibits signs of dehydration; A patient experiences significant weight loss |  |  |
|  | I would consider DISCONTINUATION of the Dato-DXd treatment regimen upon seeing the following symptoms: A patient reports moderate oral pain, ulceration, and a moderate change in diet needed without any interference in oral intake |  |  |
| **OM/S best practices – Need for Dato-DXd OM/S guidance and treatment goals** | | | |
| I believe guidance on how to manage stomatitis in patients receiving Dato-DXd treatment would help healthcare professionals and the care team improve patients’ quality of life | When determining my stomatitis prevention and treatment strategies for Dato-DXd, I would reference Everolimus guidance for best practices. |  |  |
| I believe guidance on how to prevent and manage stomatitis in patients receiving Dato-DXd treatment would help improve clinical outcomes and mitigate dose reductions, treatment delays/interruptions, or treatment discontinuation |  |  |  |
| My goals when managing stomatitis in patients receiving Dato-DXd include: preventing or reducing incidence of stomatitis, managing pain associated with stomatitis, preventing complications associated with stomatitis, maintaining the patient’s oral function (i.e., ability to open mouth, speak, swallow, etc.), optimizing the patient's quality of life, and limiting dose reductions, delays, or treatment interruptions |  |  |  |
| **OM/S best practices – General OM/S management strategies** | | | |
| I believe that educating care team members who spend significant time with the patients (e.g., nurses) on the prevention, identification, and management of stomatitis is important |  |  |  |
| I would discuss the patient’s stomatitis presentation and management strategies with the care team prior to stomatitis management initiation |  |  |  |
| Stomatitis management should be adapted to the individual patient due to variability in presentation and differing levels of pain tolerance |  |  |  |
| I would discuss prophylactic and management approaches for managing stomatitis with my patient to determine which approaches they are comfortable with and can access |  |  |  |
| **OM/S best practices – OM/S management in Dato-DXd** | | | |
| When making decisions on the management of stomatitis or potential changes to the Dato-DXd treatment regimen, the patient’s quality of life should be a key consideration |  |  |  |
| Prior to each Dato-DXd infusion, I would ask the patient about their stomatitis prophylaxis / treatment adherence and quality of life to inform if other stomatitis interventions are needed |  |  |  |
| I would evaluate the following patient outcomes while managing patients experiencing stomatitis while receiving Dato-DXd treatment: Pain level, weight, nutritional status, and quality of life |  |  |  |
| Given adequate guidance on the management of stomatitis in patients receiving Dato-DXd treatment, I would consider the risk of stomatitis as a manageable adverse event |  |  |  |
| Given adequate guidance on the management of stomatitis in patients receiving Dato-DXd treatment, I would be comfortable prescribing Dato-DXd to my patients |  |  |  |
| Based on my clinical experience, I believe stomatitis seen in patients receiving Dato-DXd treatment can be adequately controlled with the preventive and treatment measures outlined in this study |  |  |  |

##

## Supplementary Table 3 Results from the first survey, full Likert breakdown

|  | **B01** | **B02** | **B03** | **B04** | **B05** | **B06** | **L01** | **L02** | **L03** | **L04** | **L05** | **L06** | **L07** | **L08** | **OS** | **# Responses** |
| --- | --- | --- | --- | --- | --- | --- | --- | --- | --- | --- | --- | --- | --- | --- | --- | --- |
| **Description of OM/S in oncology** | | | | | | | | | | | | | | | | |
| **1. Stomatitis is a known adverse event associated with several oncology treatments for multiple cancer types, including NSCLC and breast cancer** | 9 | 9 | 9 | 8 | 9 | 9 | 7 | 9 | 9 | 8 | 3 | 8 | 7 | 7 | 9 | 15 |
| **2. In my experience, stomatitis can have a significant impact on patient quality of life and should be managed appropriately in patients receiving anticancer treatment** | 9 | 9 | 9 | 8 | 8 | 9 | 9 | 9 | 9 | 8 | 8 | 9 | 9 | 9 | 9 | 15 |
| **3. In my experience, complications associated with stomatitis can generally be mitigated when adhering to preventive, treatment, and management guidelines** | 7 | 8 | 7 | 8 | 8 | 8 | 3 | 9 | 9 | 8 | 4 | 8 | 7 | 7 | 7 | 15 |
| **4. In my experience, poorly managed stomatitis can impact patient clinical outcomes, including:** | | | | | | | | | | | | | | | | |
| **a. Malnourishment** | 9 | 9 | 7 | 7 | 9 | 7 | 9 | 9 | 9 | 8 | 9 | 9 | 8 | 9 | 9 | 15 |
| **b. Weight loss** | 9 | 9 | 8 | 7 | 9 | 9 | 9 | 9 | 9 | 8 | 9 | 9 | 8 | 9 | 9 | 15 |
| **c. Dysphagia** | 9 | 9 | 9 | 6 | 9 | 9 | 9 | 9 | 9 | 8 | 9 | 9 | 8 | 9 | 9 | 15 |
| **d. Oral candidiasis** | 9 | 9 | 6 | 5 | 8 | 5 | 9 | 9 | 9 | 9 | 5 | 7 | 8 | 9 | 1 | 15 |
| **e. Herpes simplex virus (HSV) reactivation** | 9 | 8 | 5 | 5 | 8 | 1 | 9 | 5 | 9 | 5 | 5 | NA | 8 | 9 | 1 | 15 |
| **f. Other** |  | 9 |  |  |  |  |  |  |  |  |  |  |  |  |  | 15 |
| **OM/S presentation in Dato-DXd** | | | | | | | | | | | | | | | | |
| **5. The onset of stomatitis associated with Dato-DXd treatment most commonly occurs within the initial treatment cycles of Dato-DXd, however, stomatitis can appear at any point throughout the treatment** | 9 | 9 | 8 | 8 | 8 | 7 | 7 | 9 | 7 | 8 | 7 | 9 | 5 | 8 |  | 14 |
| **6. Stomatitis associated with Dato-DXd treatment can be persistent and needs close management** | 9 | 9 | 9 | 7 | 8 | 9 | 9 | 9 | 8 | 8 | 8 | 6 | 9 | 9 |  | 14 |
| **7. I have patients who do not experience stomatitis when treated with Dato-DXd** | 9 | 8 | 7 | 9 | 8 | 7 | 3 | 9 | 3 | 7 | 9 | 9 | 7 | 9 |  | 14 |
| **Need for Dato-DXd specific OM/S guidance** | | | | | | | | | | | | | | | | |
| **8. I believe guidance on how to manage stomatitis in patients receiving Dato-DXd treatment would help healthcare professionals and the care team improve patients’ quality of life** | 9 | 9 | 9 | 9 | 9 | 9 | 9 | 9 | 9 | 8 | 8 | 9 | 5 | 9 |  | 14 |
| **9. I believe guidance on how to prevent and manage stomatitis in patients receiving Dato-DXd treatment would help improve clinical outcomes and mitigate dose reductions, treatment delays/interruptions, or treatment discontinuation** | 9 | 9 | 9 | 9 | 9 | 9 | 9 | 9 | 9 | 8 | 8 | 7 | 5 | 9 |  | 14 |
| **10. My goals when managing stomatitis in patients receiving Dato-DXd include:** | | | | | | | | | | | | | | | | |
| **a. Preventing or reducing incidence of stomatitis** | 9 | 9 | 9 | 9 | 9 | 9 | 9 | 9 | 9 | 8 | 9 | 9 | 9 | 9 |  | 14 |
| **b. Managing pain associated with stomatitis** | 9 | 9 | 9 | 9 | 9 | 6 | 9 | 9 | 9 | 8 | 9 | 8 | 9 | 9 |  | 14 |
| **c. Preventing complications associated with stomatitis** | 9 | 9 | 8 | 7 | 9 | 6 | 9 | 6 | 9 | 8 | 9 | 9 | 9 | 9 |  | 14 |
| **d. Maintaining the patient’s oral function (i.e., ability to open mouth, speak, swallow, etc.)** | 9 | 9 | 8 | 7 | 9 | 9 | 9 | 9 | 9 | 9 | 9 | 9 | 9 | 9 |  | 14 |
| **e. Optimizing the patient's quality of life** | 9 | 9 | 9 | 9 | 9 | 9 | 9 | 9 | 9 | 8 | 9 | 9 | 9 | 9 |  | 14 |
| **f. Optimizing the patient's overall survival** | 9 | 9 | 6 | 7 | 8 | 9 | 3 | 9 | 9 | 6 | 9 | 8 | 7 | 9 |  | 14 |
| **g. Limiting dose reductions, delays, or treatment interruptions** | 9 | 9 | 7 | 8 | 9 | 9 | 9 | 7 | 9 | 7 | 9 | 6 | 5 | 9 |  | 14 |
| **h. Other: please specify and rate on scale** | | | | | | | | | | | | | | | | |
| **Section 2: Guidance on the prevention, diagnosis, and treatment of OM/S in patients receiving Dato-DXd** | | | | | | | | | | | | | | | | |
| **Prevention measures for OM/S** | | | | | | | | | | | | | | | | |
| **11. I would educate my patients on the following issues prior to Dato-DXd treatment initiation:** | | | | | | | | | | | | | | | | |
| **a. Stomatitis awareness** | 9 | 9 | 9 | 8 | 9 | 9 | 9 | 9 | 9 | 8 | 7 | 8 | 8 | 9 | 9 | 15 |
| **b. Oral care (e.g., brushing and flossing teeth, dietary changes)** | 5 | 8 | 7 | 8 | 8 | 9 | 9 | 9 | 9 | 9 | 7 | 8 | 8 | 9 | 9 | 15 |
| **c. Preventive measures (e.g., mouthwashes)** | 9 | 9 | 9 | 8 | 9 | 9 | 9 | 9 | 9 | 9 | 7 | 8 | 8 | 9 | 9 | 15 |
| **d. Early recognition & monitoring of symptoms** | 7 | 9 | 9 | 8 | 9 | 9 | 9 | 9 | 9 | 9 | 7 | 8 | 8 | 9 | 9 | 15 |
| **e. Other: please specify and rate on scale** | 8 | 9 |  |  |  |  |  | 9 |  |  |  |  |  |  |  | 15 |
| **12. I would recommend a comprehensive professional oral examination by an oral health professional at Dato-DXd treatment initiation** | 1 | 3 | 4 | 7 | 5 | 1 | 1 | 2 | 7 | 3 | 6 | 8 | 3 | 5 | 1 | 15 |
| **13. Before the first cycle of Dato-DXd treatment and continuing throughout the treatment, I would prescribe a set of preventive measures to reduce the likelihood and severity of stomatitis** | 9 | 9 | 9 | 9 |  | 9 | 9 | 9 | 9 | 8 | 9 | 9 | 7 | 8 | 5 | 14 |
| **14. As part of my stomatitis prevention strategy, I would recommend behavioral and additional prophylactic oral care to my patients prior to the first cycle and continuing throughout Dato-DXd treatment, including:** | | | | | | | | | | | | | | | | |
| **a. Brushing their teeth twice daily after meals and bedtime with a soft toothbrush** | 9 | 9 | 7 | 4 | 8 | 5 | 9 | 9 | 9 | 8 | 7 | 8 | 5 | 9 | 9 | 15 |
| **b. Flossing their teeth once daily** | 4 | 9 | 7 | 4 | 8 | 5 | 9 | 9 | 9 | 9 | 5 | 8 | 5 | 9 | 9 | 15 |
| **c. Rinsing their mouth with tap water** | 4 | NA | 5 | 4 | 8 | 5 | 9 | 4 | 9 | 9 | 8 | 5 | 5 | 9 | 9 | 14 |
| **d. Daily rinse with a saline solution** | 9 | NA | 5 | 5 | 8 | 3 | 9 | 4 | 5 | 9 | 8 | 8 | 5 | 9 | 5 | 14 |
| **e. Daily rinse with a sodium bicarbonate solution** | 9 | 9 | 7 | 5 | 9 | 5 | 9 | 9 | NA | 9 | 8 | 8 | 5 | 8 | 5 | 15 |
| **f. Daily use of a prophylactic non-steroidal anti-inflammatory mouthwash (e.g., benzydamine)** | 4 | 5 | 6 | 7 | 9 | 3 | NA | 4 | NA | 9 | 8 | 8 | 5 | 5 | 1 | 15 |
| **g. Daily use of a prophylactic steroid-containing mouthwash (e.g., dexamethasone oral solution or a similar steroid)** | 9 | 9 | 9 | 8 | 9 | 9 | 9 | 9 | 9 | 9 | 8 | 8 | 8 | 5 | 1 | 15 |
| **h. Prophylactic cryotherapy (ice chips or ice water held in the patient’s mouth throughout the infusion)** | 7 | 9 | 6 | 7 | 8 | 3 | 9 | 9 | NA | 9 | 8 | 9 | 5 | 9 | 1 | 15 |
| **i. Zinc supplements** | 2 | 3 | 4 | 4 | 4 | 5 | NA | 4 | 1 | 5 | 5 | 5 | 5 | 5 | 1 | 15 |
| **j. Oral glutamine** | 2 | 5 | 4 | 4 | 4 | 3 | NA | 4 | 1 | 5 | 5 | 5 | 5 | 5 | 1 | 15 |
| **k. Honey** | 2 | 5 | 4 | 4 | 4 | 3 | NA | 4 | 1 | 5 | 5 | 5 | 5 | 5 | 1 | 15 |
| **l. Palifermin (recombinant human keratinocyte treatment)** | 3 | 3 | 4 | 4 | 4 | 6 | NA | 4 | 1 | 7 | 5 | 5 | 5 | 5 | 1 | 15 |
| **m. Antibiotics and antifungals** | 2 | 8 | 4 | 4 | 7 | 1 | NA | 4 | 1 | 5 | 4 | 2 | 3 | 5 | 1 | 15 |
| **15. I would continue preventive measures throughout the Dato-DXd treatment and on top of any treatment measures prescribed to manage stomatitis of any grade** | 9 | 9 | 9 | 8 | 8 | 7 | 9 | 8 | 9 | 8 | 6 | 8 | 9 | 8 | 9 | 15 |
| **Diagnostic measures for OM/S** | | | | | | | | | | | | | | | | |
| **16. Upon Dato-DXd treatment initiation, I would encourage patients to perform regular self-checks to identify the onset of stomatitis and immediately report any signs or symptoms** | 7 | 8 | 7 | 7 | 9 | 1 | 9 | 9 | 9 | 8 | 8 | 9 | 8 | 9 | 9 | 15 |
| **17. I would use the following as primary methods of diagnosing stomatitis and staging during the Dato-DXd regimen** | | | | | | | | | | | | | | | | |
| **a. Mouth examination prior to each infusion** | 9 | 9 | 7 | 8 | 5 | 7 | 9 | 9 | 9 | 9 | 7 | 7 | 8 | 9 | 9 | 15 |
| **b. Patient-reported symptoms** | 9 | 9 | 9 | 8 | 9 | 9 | 9 | 9 | 9 | 9 | 7 | 8 | 8 | 9 | 9 | 15 |
| **c. Bacterial, fungal or viral cultures** | 2 | 5 | 1 | 6 | 5 | 1 | 3 | 2 | 7 | 3 | 5 | 2 | 3 | 3 | 1 | 15 |
| **d. Other: please specify and rate on scale** |  |  |  |  |  |  |  | 9 | NA |  |  |  |  |  |  | 15 |
| **18. Upon the first signs of stomatitis, I would consult with an oral health specialist to confirm the diagnosis** | 2 | 3 | 4 | 7 |  | 1 | 1 | 1 | 6 | 3 | 3 | 9 | 3 | 3 | 1 | 14 |
| **Treatment approaches for mild OM/S** | | | | | | | | | | | | | | | | |
| **19. Based on current guidelines for stomatitis management, I would consider the following treatments for mild stomatitis in patients receiving Dato-DXd treatment:** | | | | | | | | | | | | | | | | |
| **a. 2% viscous lidocaine** | 8 | 7 | 4 | 8 | 9 | 9 | 7 | 7 | 9 | 7 | 5 | 8 | 7 | 8 | 9 | 15 |
| **b. Cryotherapy** | 2 | NA | 4 | 7 | 9 | 4 | 8 | 7 | 1 | 7 | 5 | 5 | 3 | 2 | 1 | 14 |
| **c. Chlorhexidine** | 5 | 9 | 4 | 6 | 9 | 6 | NA | 1 | 1 | 5 | 5 | 5 | 7 | 5 | 1 | 15 |
| **d. Topical NSAIDs** | 5 | 7 | 5 | 7 | 9 | 3 | NA | 1 | 9 | 6 | 7 | 7 | 5 | 5 | 1 | 15 |
| **e. Steroid containing mouth rinse** | 9 | 9 | 9 | 8 | 9 | 9 | 8 | 7 | 9 | 7 | 7 | 7 | 7 | 8 | 1 | 15 |
| **f. Topical corticosteroids** | 8 | 3 | NA | 8 | 9 | 9 | 8 | 2 | 5 | 7 | 7 | 7 | 7 | 5 | 1 | 15 |
| **g. Sugarless chewing gum, salivery substitutes, or sialagogues** | 5 | 5 | 7 | 6 | 8 | 1 | 9 | 1 | 1 | 7 | 7 | 8 | 5 | 5 | 1 | 15 |
| **h. Magic mouthwash** | 9 | 8 | 9 | 8 | 8 | 9 | 7 | 5 | 9 | 7 | 8 | 8 | 7 | 8 | 9 | 15 |
| **20. I would not reduce the Dato-DXd dose or interrupt the treatment regimen for my patients receiving Dato-DXd treatment who present with a mild clinical presentation of stomatitis** | 8 | 8 | 7 | 7 | 9 | 9 | 3 | 9 | 9 | 3 | 2 | 3 | 9 | 6 | 5 | 15 |
| **Treatment approaches for moderate OM/S** | | | | | | | | | | | | | | | | |
| **21. Based on current guidelines for stomatitis management, I would consider the following treatments for moderate stomatitis in patients receiving Dato-DXd treatment in addition to treatments considered for mild stomatitis:** | | | | | | | | | | | | | | | | |
| **a. Intralesional steroid injections** | 6 | 5 | 1 | 5 | 3 | 3 | NA | 2 | NA | 3 | 5 | 4 | 5 | 5 | 1 | 15 |
| **b. Topically applied aloe** | 5 | 5 | 1 | 6 | 5 | 5 | NA | 2 | NA | 4 | 5 | 6 | 5 | 5 | 1 | 15 |
| **c. Mucosal coating agents** | 9 | 5 | 7 | 6 |  | 5 | 8 | 2 | NA | 4 | 5 | 7 | 7 | 5 | 1 | 14 |
| **d. Systemic opioids** | 8 | 8 | 9 | 7 |  | 9 | 7 | 9 | 9 | 7 | 5 | 7 | 7 | 8 | 9 | 14 |
| **e. Antibiotics or antifungals** | 6 | 7 | 7 | 6 | 5 | 7 | NA | 9 | 5 | 7 | 7 | 1 | 7 | 7 | 1 | 15 |
| **f. Magic mouthwash** | 9 | 8 | 9 | 8 | 8 | 9 | 7 | 5 | 9 | 7 | 7 | 9 | 7 | 9 | 9 | 15 |
| **g. Other** |  |  |  |  |  |  |  |  |  |  |  |  |  |  |  |  |
| **22. For patients experiencing moderate stomatitis while receiving Dato-DXd treatment, I would consider:** | | | | | | | | | | | | | | | | |
| **a. Keeping the Dato-DXd treatment regimen as is** | 3 | 3 | 4 | 7 | 5 | 3 | 2 | 1 | 1 | 6 | 4 | 3 |  | 5 | 6 | 14 |
| **b. Delaying/interrupting Dato-DXd treatment** | 9 | 8 | 9 | 8 | 8 | 8 | 9 | 9 | 7 | 7 | 4 | 8 |  | 6 | 6 | 14 |
| **c. Reducing Dato-DXd dose** | 8 | 8 | 7 | 9 | 7 | 5 | 9 | 9 | 9 | 7 | 4 | 8 |  | 5 | 6 | 14 |
| **d. Discontinuing Dato-DXd treatment** | 2 | 1 | 4 | 5 | 1 | 1 | 5 | 1 | 1 | 7 | 4 | 1 |  | 1 | 5 | 14 |
| **e. Other consideration** | 8 |  |  |  |  |  |  |  |  | a,b,c |  |  | 7 |  | 5 | 15 |
| **Treatment approaches for severe OM/S** | | | | | | | | | | | | | | | | |
| **23. Based on current guidelines for stomatitis management, I would consider the following treatments for severe stomatitis in patients receiving Dato-DXd treatment in addition to treatments considered for mild or moderate stomatitis:** | | | | | | | | | | | | | | | | |
| **a. Systemic oral corticosteroids** | 5 | 5 | 9 | 8 | 5 |  | 7 | 9 | 9 | 7 | 7 | 8 | 7 | 9 | 1 | 14 |
| **b. Daily low-level laser therapy** | 5 | 3 | N/A | 6 | 2 |  | N/A | 1 | N/A | 2 | 5 | 5 | 5 | 5 | 5 | 14 |
| **c. Systemic opioids** | 9 | 7 | 9 | 8 | 5 |  |  | 9 | 9 | 7 | 7 | 8 | 7 | 9 | 8 | 13 |
| **d. Others** | 8 |  |  |  |  |  |  |  |  |  |  |  |  |  |  | 15 |
| **24. For patients experiencing severe stomatitis while receiving Dato-DXd treatment, I would consider:** | | | | | | | | | | | | | | | | |
| **a. Keeping the Dato-DXd treatment regimen as is** | 1 | 1 | 1 | 3 | 1 | 1 | 1 | 1 | 1 | 2 | 2 | 1 | 3 | 1 |  | 14 |
| **b. Delaying/interruption Dato-DXd treatment** | 9 | 9 | 9 | 9 | 9 | 9 | 9 | 9 | 9 | 8 | 2 | 9 | 8 | 9 |  | 14 |
| **c. Reducing Dato-DXd dose** | 9 | 9 | 9 | 9 | 9 | 9 | 4 | 9 | 9 | 8 | 2 | 9 | 8 | 9 |  | 14 |
| **d. Discontinuing Dato-DXd treatment** | 4 | 9 | 9 | 7 | 5 | 1 | 9 | 5 | 8 | 9 | 8 | 4 | 8 | 4 |  | 14 |
| **e. Other** | 9 |  |  |  |  |  |  |  |  |  |  |  |  |  |  | 15 |
| **Section 3: OM/S patient management** | | | | | | | | | | | | | | | | |
| **Best practices for managing patients with OM/S** | | | | | | | | | | | | | | | | |
| **25. I believe that educating care team members who spend significant time with the patients (e.g., nurses) on the prevention, identification, and management of stomatitis is important** | 9 | 9 | 9 | 8 | 9 | 9 | 9 | 9 | 9 | 8 | 9 | 9 | 7 | 9 | 9 | 15 |
| **26. I would discuss the patient’s stomatitis presentation and management strategies with the care team prior to stomatitis management initiation** | 9 | 9 | 9 | 7 | 5 | 9 | 9 | 9 | 9 | 8 | 9 | 7 | 7 | 9 | 9 | 15 |
| **27. When making decisions on the management of stomatitis or potential changes to the Dato-DXd treatment regimen, the patient’s quality of life should be a key consideration** | 9 | 9 | 9 | 8 | 9 | 9 | 8 | 9 | 9 | 9 | 9 | 9 | 9 | 9 | 9 | 15 |
| **28. Prior to each Dato-DXd infusion, I would ask the patient about their stomatitis prophylaxis / treatment adherence and quality of life to inform if other stomatitis interventions are needed** | 9 | 9 | 9 | 8 | 9 | 9 | 9 | 9 | 9 | 8 | 9 | 9 | 9 |  | 9 | 14 |
| **29. I would evaluate the following patient outcomes while managing patients experiencing stomatitis while receiving Dato-DXd treatment:** | | | | | | | | | | | | | | | | |
| **a. Pain level** | 9 | 9 | 9 | 9 | 9 | 9 | 9 | 9 | 9 | 9 | 9 | 7 | 8 | 9 | 9 | 15 |
| **b. Weight** | 9 | 9 | 9 | 8 | 8 | 9 | 9 | 9 | 9 | 9 | 9 | 6 | 8 | 9 | 9 | 15 |
| **c. Nutritional status** | 9 | 9 | 9 | 8 | 5 | 9 | 9 | 9 | 9 | 9 | 9 | 6 | 8 | 9 | 9 | 15 |
| **d. Quality of life** | 9 | 9 | 9 | 9 | 9 | 9 | 9 | 9 | 9 | 9 | 8 | 9 | 8 | 9 | 9 | 15 |
| **e. Other** | 9 |  |  |  |  |  |  | 9 |  |  |  |  |  |  |  | 15 |
| **30. Stomatitis management should be adapted to the individual patient due to variability in presentation and differing levels of pain tolerance** | 7 | 7 | 8 | 9 | 9 | 9 | 9 | 5 | 9 | 9 | 9 | 8 | 8 | 9 | 9 | 15 |
| **31. I would discuss prophylactic and management approaches for managing stomatitis with my patient to determine which approaches they are comfortable with and can access** | 9 | 3 | 9 | 9 | 9 | 9 | 9 | 8 | 9 | 9 | 9 | 7 | 8 | 9 | 9 | 15 |
| **Management of OM/S with Dato-DXd** | | | | | | | | | | | | | | | | |
| **32. Given adequate guidance on the management of stomatitis in patients receiving Dato-DXd treatment, I would consider the risk of stomatitis as a manageable adverse event** | 9 | 8 | 9 | 8 | 9 | 9 | 7 | 9 | 9 | 8 | 4 | 8 | 7 | 9 | 7 | 15 |
| **33. Given adequate guidance on the management of stomatitis in patients receiving Dato-DXd treatment, I would be comfortable prescribing Dato-DXd to my patients** | 9 | 9 | 9 | 9 | 9 | 9 | 9 | 9 | 9 | 8 | 5 | 9 | 7 | 9 |  | 14 |
| **34. Based on my clinical experience, I believe stomatitis seen in patients receiving Dato-DXd treatment can be adequately controlled with the preventive and treatment measures outlined in this study** | 9 | 9 | 7 | 8 | 9 | 8 | 4 | 9 | 9 | 8 | 4 | 7 | 7 | 9 |  | 14 |

NA = not answered, L = lung caner expert, B = breast cancer expert, OS = oral medicine specialist

## Supplementary Table 4 Results from the second survey, full Likert breakdown

|  | **OS** | **B01** | **B03** | **B04** | **B05** | **B06** | **L01** | **L02** | **L03** | **L04** | **L05** | **L06** | **L07** | **L08** |
| --- | --- | --- | --- | --- | --- | --- | --- | --- | --- | --- | --- | --- | --- | --- |
| **1a. When determining if a patient has stomatitis, a physical examination and patient-reported symptoms are sufficient to make a diagnosis.** | 9 | 8 | 8 | 9 | 8 | 8 | 8 | 8 | 8 | 9 | 9 | 9 | 8 | 9 |
| **2. As part of my prophylactic strategy to reduce the likelihood and severity of stomatitis, I would recommend ‘do no harm’ behavioral changes for my patient, including:** | | | | | | | | | | | | | | |
| **a. Teeth brushing -** | 9 | 7 | 9 | 9 | 9 | 9 | 8 | 9 | 3 | 9 | 9 |  | 8 | 8 |
| **b. Flossing -** | 9 | 7 | 5 | 9 | 9 | 7 | 8 | 9 | 3 | 9 | 9 |  | 8 | 5 |
| **c. Rinsing their mouth with water** | 9 | 7 | 9 | 9 | 9 | 9 | 8 | 6 | 5 | 9 | 9 |  | 8 | 8 |
| **d. Avoiding acidic or crunchy foods** | 5 | 7 | 9 | 9 | 9 | 7 | 7 | 6 | 8 | 7 | 9 |  | 8 | 7 |
| **e. Cryotherapy (ice chips or ice water held in the patient's mouth)** | 1 | 8 | 9 | 9 | 9 | 7 | 8 | 8 |  | 9 | 9 |  | 8 | 7 |
| **f. Rinsing their mouth with a bicarbonate solution** | 5 | 8 | 7 | 9 | 9 | 7 | 5 | 8 | 8 | 7 | 9 |  | 8 | 7 |
| **6. I believe that only Dato-DXd patients with the following attributes should receive a prophylactic regimen to reduce the likelihood and severity of stomatitis:** | | | | | | | | | | | | | | |
| **Patients with a prior history of mouth sores** |  |  |  |  | x |  |  |  |  |  |  | x |  |  |
| **Patients aged 65+ years old** |  |  |  |  | x |  |  |  |  |  |  | x |  |  |
| **Patients with poor nutritional status** |  |  |  |  | x |  |  |  |  |  |  | x |  |  |
| **Patients with poor oral hygiene or other oral conditions** |  |  |  |  | x |  |  |  |  |  |  | x |  |  |
| **Patients with a prior history of smoking** |  |  |  |  |  |  |  |  |  |  |  | x |  |  |
| **All patients starting Dato-DXd** | x | x | x | x | x | x | x | x | x | x | x | x | x | x |
| **7. For patients I would place on a prophylactic regimen, I would begin prophylaxis:** | | | | | | | | | | | | | | |
| **Prior to Dato-DXd treatment initiation** | x |  |  |  |  |  | x |  | x |  |  | x |  |  |
| **At Dato-DXd treatment initiation** |  | x | x | x | x | x |  |  |  | x | x |  | x |  |
| **After Dato-DXd treatment initiation, in patients WITHOUT stomatitis symptoms** |  |  |  |  |  |  |  |  |  |  |  |  |  |  |
| **After Dato-DXd treatment initiation, in patients WITH stomatitis symptoms** |  |  |  |  |  |  |  | x |  |  |  |  |  | x |
| **8. As part of my prophylactic and ongoing management strategy of stomatitis, I would recommend a dexamethasone mouth rinse to my Dato-DXd patients** | 1 | 8 | 9 | 9 | 9 | 9 | 9 | 8 | 9 | 7 | 9 | 9 | 9 | 6 |
| **9. I would recommend my Dato-DXd patients use the dexamethasone mouth rinse with the following frequency:** | | | | | | | | | | | | | | |
| **1x per day** |  |  |  |  |  |  |  |  |  |  |  |  |  |  |
| **2x per day** |  |  |  |  |  |  | x | x |  |  |  | x | x | x |
| **3x per day** |  | x | x |  |  |  |  |  |  | x |  |  |  |  |
| **4x per day** |  |  |  | x | x | x |  |  | x |  | x |  |  |  |
| **I would not recommend a dexamethasone mouth rinse to my Dato-DXd patients** | x |  |  |  |  |  |  |  |  |  |  |  |  |  |
| **10. I would recommend my Dato-DXd patients swish the dexamethasone mouth rinse in their mouth for the following period of time before discarding:** | | | | | | | | | | | | | | |
| **Under 1 minute** |  |  |  |  |  |  |  |  |  |  |  |  | x | x |
| **1 minute** |  |  | x | x |  |  | x | x |  |  |  | x |  |  |
| **1-2 minutes** |  | x |  |  | x | x |  |  | x |  |  |  |  |  |
| **2-3 minutes** |  |  |  |  |  |  |  |  |  | x | x |  |  |  |
| **I would not recommend a dexamethasone mouth rinse to my Dato-DXd patients** | x |  |  |  |  |  |  |  |  |  |  |  |  |  |
| **11. If dexamethasone rinse is not available, I would recommend another steroid mouth rinse which does not contain alcohol to my Dato-DXd patients.** | 1 | 8 | 9 | 9 | 9 | 9 | 6 | 5 | 9 | 7 | 9 | 9 |  | 7 |
| **12. In the absence of a steroid mouth rinse, I would recommend a bland (i.e., non-alcoholic and/or bicarbonate-containing) mouth rinse to my Dato-DXd patients.** | 1 | 8 | 9 | 9 | 9 | 9 | 8 | 6 | 9 | 7 | 9 | 9 | 8 | 7 |
| **15. [Breast Cancer Experts only] When determining my stomatitis prevention and treatment strategies for Dato-DXd, I would reference Everolimus guidance for best practices. Guidance includes: Dexamethasone mouth rinse (10 ml, 0.5mg/5ml solution) 4x/day, 2 minutes per time** |  | 9 | 9 | 9 | 9 | 9 |  |  |  |  |  |  |  |  |
| **16. For the following Dato-DXd patients, I would monitor for signs of stomatitis at the following frequency: [Please select one option per stomatitis severity]** | | | | | | | | | | | | | | |
| **Non-symptomatic - 1x per treatment cycle (3 weeks)** |  | x | x |  |  | x | x | x | x | x | x | x | x |  |
| **Non-symptomatic - 1x per every 2 treatment cycles (6 weeks)** |  |  |  | x |  |  |  |  |  |  |  |  |  | x |
| **Non-symptomatic - Every 6 months** |  |  |  |  |  |  |  |  |  |  |  |  |  |  |
| **Non-symptomatic - Once a year** |  |  |  |  |  |  |  |  |  |  |  |  |  |  |
| **Non-symptomatic - I would only monitor once symptoms arise** | x |  |  |  | x |  |  |  |  |  |  |  |  |  |
| **Mild presentation of stomatitis - 1x per treatment cycle (3 weeks)** | x | x | x | x | x | x | x | x | x | x | x | x | x |  |
| **Mild presentation of stomatitis - 1x per every 2 treatment cycles (6 weeks)** |  |  |  |  |  |  |  |  |  |  |  |  |  | x |
| **Mild presentation of stomatitis - Every 6 months** |  |  |  |  |  |  |  |  |  |  |  |  |  |  |
| **Mild presentation of stomatitis - Once a year** |  |  |  |  |  |  |  |  |  |  |  |  |  |  |
| **Mild presentation of stomatitis - I would only monitor once symptoms arise** |  |  |  |  |  |  |  |  |  |  |  |  |  |  |
| **Moderate presentation of stomatitis - 1x per treatment cycle (3 weeks)** | x | x | x | x | x | x | x | x | x | x | x | x | x |  |
| **Moderate presentation of stomatitis - 1x per every 2 treatment cycles (6 weeks)** |  |  |  |  |  |  |  |  |  |  |  |  |  | x |
| **Moderate presentation of stomatitis - Every 6 months** |  |  |  |  |  |  |  |  |  |  |  |  |  |  |
| **Moderate presentation of stomatitis - Once a year** |  |  |  |  |  |  |  |  |  |  |  |  |  |  |
| **Moderate presentation of stomatitis - I would only monitor once symptoms arise** |  |  |  |  |  |  |  |  |  |  |  |  |  |  |
| **Severe presentation of stomatitis - 1x per treatment cycle (3 weeks)** | x | x | x | x | x | x | x | x | x | x | x | x | x |  |
| **Severe presentation of stomatitis - 1x per every 2 treatment cycles (6 weeks)** |  |  |  |  |  |  |  |  |  |  |  |  |  | x |
| **Severe presentation of stomatitis - Every 6 months** |  |  |  |  |  |  |  |  |  |  |  |  |  |  |
| **Severe presentation of stomatitis - Once a year** |  |  |  |  |  |  |  |  |  |  |  |  |  |  |
| **Severe presentation of stomatitis - I would only monitor once symptoms arise** |  |  |  |  |  |  |  |  |  |  |  |  |  |  |
| **18. I would recommend increasing the prophylactic and on-going stomatitis management strategies and NOT alter Dato-DXd treatment regiment upon seeing the following symptoms:** | | | | | | | | | | | | | | |
| **a. A patient reports mild oral pain, no ulceration, and limited* change in diet needed without any interference in oral intake*Includes avoiding spicy or acidic food** | 9 | 8 | 9 | 9 | 9 | 8 | 1 | 8 | 8 | 7 | 9 | 9 | 8 | 6 |
| **b. A patient reports mild oral pain, slight ulceration, and limited* change in diet needed without any interference in oral intake*Includes avoiding spicy or acidic food** | 9 | 7 | 9 | 9 | 9 | 8 | 4 | 7 | 8 | 7 | 5 | 9 | 4 | 7 |
| **c. A patient reports mild oral pain, slight ulceration, and some* change in diet needed without any interference in oral intake*Some solid foods may be limited and some change to diet may be required** | 9 | 7 | 9 | 7 | 9 | 8 | 7 | 6 | 8 | 5 | 1 | 9 | 2 | 6 |
| **A patient reports moderate oral pain, ulceration, and a moderate change in diet needed without any interference in oral intake** | 9 | 3 | 7 | 3 | 9 | 2 | 9 | 5 | 8 | 3 | 1 | 1 | 1 | 7 |
| **e. A patient reports severe oral pain, ulceration, and significant interference with oral intake** | 5 | 3 | 1 | 1 | 9 | 1 | 9 | 2 | 8 | 1 | 1 | 1 | 1 | 7 |
| **19. I would consider DELAYING Dato-DXd treatment regimen upon seeing the following symptoms:** |  |  |  |  |  |  |  |  |  |  |  |  |  |  |
| **a. A patient reports mild oral pain, no ulceration, and limited* change in diet needed without any interference in oral intake*Includes avoiding spicy or acidic food** | 1 | 2 | 3 | 1 | 2 | 2 | 1 | 7 | 3 | 1 | 3 | 1 | 8 | 5 |
| **b. A patient reports mild oral pain, slight ulceration, and limited* change in diet needed without any interference in oral intake*Includes avoiding spicy or acidic food** | 1 | 2 | 8 | 3 | 2 | 2 | 3 | 8 | 3 | 1 | 5 | 1 | 5 | 5 |
| **c. A patient reports mild oral pain, slight ulceration, and some* change in diet needed without any interference in oral intake*Some solid foods may be limited and some change to diet may be required** | 1 | 2 | 3 | 5 | 7 | 2 | 4 | 9 | 3 | 5 | 9 | 1 | 3 | 6 |
| **d. A patient reports moderate oral pain, ulceration, and a moderate change in diet needed without any interference in oral intake** | 1 | 8 | 9 | 9 | 9 | 9 | 6 | 9 | 3 | 7 | 9 | 7 | 2 | 7 |
| **e. A patient reports severe oral pain, ulceration, and significant interference with oral intake** | 9 | 8 |  | 9 | 9 | 9 |  | 9 | 8 | 9 | 9 | 9 | 1 | 7 |
| **f. A patient exhibits signs of dehydration** | 5 | 8 |  | 9 | 9 | 9 |  | 9 | 8 | 9 | 9 | 9 | 1 | 8 |
| **g. A patient experiences significant weight loss** | 5 | 8 |  | 9 | 9 | 9 |  | 9 | 8 | 9 | 9 | 9 | 1 | 8 |
| **h. A patient has severe pain and is hospitalized for stabilization and/or pain control** | 5 | 8 |  | 9 | 9 | 9 |  | 9 | 8 | 9 | 9 | 9 | 1 |  |
| **20. I would consider REDUCING DOSAGE of Dato-DXd treatment regimen upon seeing the following symptoms:** | | | | | | | | | | | | | | |
| **a. A patient reports mild oral pain, no ulceration, and limited* change in diet needed without any interference in oral intake*Includes avoiding spicy or acidic food** | 1 | 7 | 2 | 1 | 1 | 2 | 3 | 2 | 3 | 1 | 2 | 1 | 8 | 2 |
| **b. A patient reports mild oral pain, slight ulceration, and limited* change in diet needed without any interference in oral intake*Includes avoiding spicy or acidic food** | 1 | 6 | 2 | 2 | 1 | 2 | 3 | 3 | 3 | 1 | 5 | 1 | 8 | 2 |
| **c. A patient reports mild oral pain, slight ulceration, and some* change in diet needed without any interference in oral intake*Some solid foods may be limited and some change to diet may be required** | 1 | 6 | 3 | 3 | 1 | 5 | 5 | 6 | 3 | 1 | 9 | 2 | 5 | 4 |
| **d. A patient reports moderate oral pain, ulceration, and a moderate change in diet needed without any interference in oral intake** |  |  | 8 | 7 | 7 |  | 5 | 7 | 3 | 3 | 9 | 7 | 3 | 6 |
| **e. A patient reports severe oral pain, ulceration, and significant interference with oral intake** |  |  | 9 | 9 | 9 |  |  | 9 | 5 | 9 | 9 | 9 | 1 | 6 |
| **f. A patient exhibits signs of dehydration** |  |  | 9 | 9 | 9 |  |  | 9 | 5 | 9 | 9 | 9 | 1 |  |
| **g. A patient experiences significant weight loss** |  |  | 9 | 9 | 9 |  |  | 9 | 8 | 9 | 9 | 9 | 1 |  |
| **h. A patient has severe pain and is hospitalized for stabilization and/or pain control** | 5 |  | 9 | 9 | 9 | 8 |  | 9 | 8 | 9 |  | 9 | 1 | 8 |
| **21. I would consider DISCONTINUATION of the Dato-DXd treatment regimen upon seeing the following symptoms:** | | | | | | | | | | | | | | |
| **a. A patient reports moderate oral pain, ulceration, and a moderate change in diet needed without any interference in oral intake** | 1 | 2 | 1 | 1 | 1 | 1 | 1 | 3 |  | 1 | 2 | 3 | 5 | 1 |
| **b. A patient reports severe oral pain, ulceration, and significant interference with oral intake** | 5 | 5 | 3 | 5 | 7 | 1 |  | 8 |  | 3 | 2 | 8 | 9 | 7 |
| **c. A patient exhibits signs of dehydration** | 5 | 5 | 3 | 3 | 8 | 5 |  | 9 |  | 3 | 2 | 9 | 9 | 6 |
| **d. A patient experiences significant weight loss** | 5 | 5 | 8 | 3 | 9 | 5 |  | 9 | NA | 3 | 2 | 9 | 9 | 7 |
| **e. A patient has severe pain and is hospitalized for stabilization and/or pain control** | 5 | 9 | 9 | 5 | 9 | 8 | 7 | 9 | 8 | 7 | 8 | 9 | 9 | 7 |

Legend:

NA = not answered, L = lung caner expert, B = breast cancer expert, OS = oral medicine specialist

## Supplementary Table 5 Results from the first survey, summary of scoring

|  | **Average score** | **#agree** | **% agree** | **# of neither nor** | **% neither nor** | **# of disagree** | **% disagree** | **# of NA** | **% of NA** |
| --- | --- | --- | --- | --- | --- | --- | --- | --- | --- |
| **Section 1: Overview of OM/S in Dato-DXd** | | | | | | | | | |
| **Description of OM/S in oncology** | | | | | | | | | |
| **1. Stomatitis is a known adverse event associated with several oncology treatments for multiple cancer types, including NSCLC and breast cancer** | 8 | 14 | 93% | 0 | 0% | 1 | 7% | 0 | 0% |
| **2. In my experience, stomatitis can have a significant impact on patient quality of life and should be managed appropriately in patients receiving anticancer treatment** | 8.733 | 15 | 100% | 0 | 0% | 0 | 0% | 0 | 0% |
| **3. In my experience, complications associated with stomatitis can generally be mitigated when adhering to preventive, treatment, and management guidelines** | 7.2 | 13 | 87% | 1 | 7% | 1 | 7% | 0 | 0% |
| **4. In my experience, poorly managed stomatitis can impact patient clinical outcomes, including:** | | | | | | | | | |
| **a. Malnourishment** | 8.467 | 15 | 100% | 0 | 0% | 0 | 0% | 0 | 0% |
| **b. Weight loss** | 8.667 | 15 | 100% | 0 | 0% | 0 | 0% | 0 | 0% |
| **c. Dysphagia** | 8.667 | 14 | 93% | 1 | 7% | 0 | 0% | 0 | 0% |
| **d. Oral candidiasis** | 7.2 | 10 | 67% | 4 | 27% | 1 | 7% | 0 | 0% |
| **e. Herpes simplex virus (HSV) reactivation** | 6.214 | 7 | 47% | 5 | 33% | 2 | 13% | 1 | 7% |
| **f. Other** | 9 | 1 | 7% | 0 | 0% | 0 | 0% | 0 | 0% |
| **OM/S presentation in Dato-DXd** | | | | | | | | | |
| **5. The onset of stomatitis associated with Dato-DXd treatment most commonly occurs within the initial treatment cycles of Dato-DXd, however, stomatitis can appear at any point throughout the treatment** | 7.786 | 13 | 93% | 1 | 7% | 0 | 0% | 0 | 0% |
| **6. Stomatitis associated with Dato-DXd treatment can be persistent and needs close management** | 8.357 | 13 | 93% | 1 | 7% | 0 | 0% | 0 | 0% |
| **7. I have patients who do not experience stomatitis when treated with Dato-DXd** | 7.429 | 12 | 86% | 0 | 0% | 2 | 14% | 0 | 0% |
| **Need for Dato-DXd specific OM/S guidance** | | | | | | | | | |
| **8. I believe guidance on how to manage stomatitis in patients receiving Dato-DXd treatment would help healthcare professionals and the care team improve patients’ quality of life** | 8.571 | 13 | 93% | 1 | 7% | 0 | 0% | 0 | 0% |
| **9. I believe guidance on how to prevent and manage stomatitis in patients receiving Dato-DXd treatment would help improve clinical outcomes and mitigate dose reductions, treatment delays/interruptions, or treatment discontinuation** | 8.429 | 13 | 93% | 1 | 7% | 0 | 0% | 0 | 0% |
| **10. My goals when managing stomatitis in patients receiving Dato-DXd include:** | | | | | | | | |  |
| **a. Preventing or reducing incidence of stomatitis** | 8.929 | 14 | 100% | 0 | 0% | 0 | 0% | 0 | 0% |
| **b. Managing pain associated with stomatitis** | 8.643 | 13 | 93% | 1 | 7% | 0 | 0% | 0 | 0% |
| **c. Preventing complications associated with stomatitis** | 8.286 | 12 | 86% | 2 | 14% | 0 | 0% | 0 | 0% |
| **d. Maintaining the patient’s oral function (i.e., ability to open mouth, speak, swallow, etc.)** | 8.786 | 14 | 100% | 0 | 0% | 0 | 0% | 0 | 0% |
| **e. Optimizing the patient's quality of life** | 8.929 | 14 | 100% | 0 | 0% | 0 | 0% | 0 | 0% |
| **f. Optimizing the patient's overall survival** | 7.714 | 11 | 79% | 2 | 14% | 1 | 7% | 0 | 0% |
| **g. Limiting dose reductions, delays, or treatment interruptions** | 8 | 12 | 86% | 2 | 14% | 0 | 0% | 0 | 0% |
| **h. Other: please specify and rate on scale** |  |  |  |  |  |  |  |  |  |
| **Section 2: Guidance on the prevention, diagnosis, and treatment of OM/S in patients receiving Dato-DXd** | | | | | | | | | |
| **Prevention measures for OM/S** | | | | | | | | | |
| **11. I would educate my patients on the following issues prior to Dato-DXd treatment initiation:** | | | | | | | | | |
| **a. Stomatitis awareness** | 8.6 | 15 | 100% | 0 | 0% | 0 | 0% | 0 | 0% |
| **b. Oral care (e.g., brushing and flossing teeth, dietary changes)** | 8.133 | 14 | 93% | 1 | 7% | 0 | 0% | 0 | 0% |
| **c. Preventive measures (e.g., mouthwashes)** | 8.667 | 15 | 100% | 0 | 0% | 0 | 0% | 0 | 0% |
| **d. Early recognition & monitoring of symptoms** | 8.533 | 15 | 100% | 0 | 0% | 0 | 0% | 0 | 0% |
| **e. Other: please specify and rate on scale** | 8.667 | 3 | 20% | 0 | 0% | 0 | 0% | 0 | 0% |
| **12. I would recommend a comprehensive professional oral examination by an oral health professional at Dato-DXd treatment initiation** | 3.8 | 3 | 20% | 4 | 27% | 8 | 53% | 0 | 0% |
| **13. Before the first cycle of Dato-DXd treatment and continuing throughout the treatment, I would prescribe a set of preventive measures to reduce the likelihood and severity of stomatitis** | 8.429 | 13 | 93% | 1 | 7% | 0 | 0% | 0 | 0% |
| **14. As part of my stomatitis prevention strategy, I would recommend behavioral and additional prophylactic oral care to my patients prior to the first cycle and continuing throughout Dato-DXd treatment, including:** | | | | | | | | | |
| **a. Brushing their teeth twice daily after meals and bedtime with a soft toothbrush** | 7.667 | 12 | 80% | 3 | 20% | 0 | 0% | 0 | 0% |
| **b. Flossing their teeth once daily** | 7.267 | 10 | 67% | 5 | 33% | 0 | 0% | 0 | 0% |
| **c. Rinsing their mouth with tap water** | 6.643 | 7 | 50% | 7 | 50% | 0 | 0% | 0 | 0% |
| **d. Daily rinse with a saline solution** | 6.571 | 7 | 50% | 6 | 43% | 1 | 7% | 0 | 0% |
| **e. Daily rinse with a sodium bicarbonate solution** | 7.5 | 10 | 67% | 4 | 27% | 0 | 0% | 1 | 7% |
| **f. Daily use of a prophylactic non-steroidal anti-inflammatory mouthwash (e.g., benzydamine)** | 5.692 | 5 | 33% | 6 | 40% | 2 | 13% | 2 | 13% |
| **g. Daily use of a prophylactic steroid-containing mouthwash (e.g., dexamethasone oral solution or a similar steroid)** | 7.933 | 13 | 87% | 1 | 7% | 1 | 7% | 0 | 0% |
| **h. Prophylactic cryotherapy (ice chips or ice water held in the patient’s mouth throughout the infusion)** | 7.071 | 10 | 67% | 2 | 13% | 2 | 13% | 1 | 7% |
| **i. Zinc supplements** | 3.786 | 0 | 0% | 10 | 67% | 4 | 27% | 1 | 7% |
| **j. Oral glutamine** | 3.786 | 0 | 0% | 10 | 67% | 4 | 27% | 1 | 7% |
| **k. Honey** | 3.786 | 0 | 0% | 10 | 67% | 4 | 27% | 1 | 7% |
| **l. Palifermin (recombinant human keratinocyte treatment)** | 4.071 | 1 | 7% | 9 | 60% | 4 | 27% | 1 | 7% |
| **m. Antibiotics and antifungals** | 3.643 | 2 | 13% | 6 | 40% | 6 | 40% | 1 | 7% |
| **15. I would continue preventive measures throughout the Dato-DXd treatment and on top of any treatment measures prescribed to manage stomatitis of any grade** | 8.267 | 14 | 93% | 1 | 7% | 0 | 0% | 0 | 0% |
| **Diagnostic measures for OM/S** | | | | | | | | | |
| **16. Upon Dato-DXd treatment initiation, I would encourage patients to perform regular self-checks to identify the onset of stomatitis and immediately report any signs or symptoms** | 7.8 | 14 | 93% | 0 | 0% | 1 | 7% | 0 | 0% |
| **17. I would use the following as primary methods of diagnosing stomatitis and staging during the Dato-DXd regimen** | | | | | | | | | |
| **a. Mouth examination prior to each infusion** | 8.067 | 14 | 93% | 1 | 7% | 0 | 0% | 0 | 0% |
| **b. Patient-reported symptoms** | 8.667 | 15 | 100% | 0 | 0% | 0 | 0% | 0 | 0% |
| **c. Bacterial, fungal or viral cultures** | 3.267 | 1 | 7% | 4 | 27% | 10 | 67% | 0 | 0% |
| **d. Other: please specify and rate on scale** | 9 | 1 | 7% | 0 | 0% | 0 | 0% | 0 | 0% |
| **18. Upon the first signs of stomatitis, I would consult with an oral health specialist to confirm the diagnosis** | 3.357 | 2 | 14% | 2 | 14% | 10 | 71% | 0 | 0% |
| **Treatment approaches for mild OM/S** | | | | | | | | | |
| **19. Based on current guidelines for stomatitis management, I would consider the following treatments for mild stomatitis in patients receiving Dato-DXd treatment:** | | | | | | | | | |
| **a. 2% viscous lidocaine** | 7.467 | 13 | 87% | 2 | 13% | 0 | 0% | 0 | 0% |
| **b. Cryotherapy** | 4.643 | 5 | 36% | 4 | 29% | 5 | 36% | 0 | 0% |
| **c. Chlorhexidine** | 4.929 | 3 | 20% | 8 | 53% | 3 | 20% | 1 | 7% |
| **d. Topical NSAIDs** | 5.5 | 6 | 40% | 5 | 33% | 3 | 20% | 1 | 7% |
| **e. Steroid containing mouth rinse** | 7.6 | 14 | 93% | 0 | 0% | 1 | 7% | 0 | 0% |
| **f. Topical corticosteroids** | 6.143 | 9 | 60% | 2 | 13% | 3 | 20% | 1 | 7% |
| **g. Sugarless chewing gum, salivery substitutes, or sialagogues** | 5.067 | 6 | 40% | 5 | 33% | 4 | 27% | 0 | 0% |
| **h. Magic mouthwash** | 7.933 | 14 | 93% | 1 | 7% | 0 | 0% | 0 | 0% |
| **20. I would not reduce the Dato-DXd dose or interrupt the treatment regimen for my patients receiving Dato-DXd treatment who present with a mild clinical presentation of stomatitis** | 6.467 | 9 | 60% | 2 | 13% | 4 | 27% | 0 | 0% |
| **Treatment approaches for moderate OM/S** | | | | | | | | | |
| **21. Based on current guidelines for stomatitis management, I would consider the following treatments for moderate stomatitis in patients receiving Dato-DXd treatment in addition to treatments considered for mild stomatitis:** | | | | | | | | | |
| **a. Intralesional steroid injections** | 3.692 | 0 | 0% | 7 | 47% | 6 | 40% | 2 | 13% |
| **b. Topically applied aloe** | 4.231 | 0 | 0% | 10 | 67% | 3 | 20% | 2 | 13% |
| **c. Mucosal coating agents** | 5.462 | 5 | 36% | 6 | 43% | 2 | 14% | 1 | 7% |
| **d. Systemic opioids** | 7.786 | 13 | 93% | 1 | 7% | 0 | 0% | 0 | 0% |
| **e. Antibiotics or antifungals** | 5.857 | 8 | 53% | 4 | 27% | 2 | 13% | 1 | 7% |
| **f. Magic mouthwash** | 8 | 14 | 93% | 1 | 7% | 0 | 0% | 0 | 0% |
| **g. Other** |  |  |  |  |  |  |  |  |  |
| **22. For patients experiencing moderate stomatitis while receiving Dato-DXd treatment, I would consider:** | | | | | | | | | |
| **a. Keeping the Dato-DXd treatment regimen as is** | 3.786 | 1 | 7% | 6 | 43% | 7 | 50% | 0 | 0% |
| **b. Delaying/interrupting Dato-DXd treatment** | 7.571 | 11 | 79% | 3 | 21% | 0 | 0% | 0 | 0% |
| **c. Reducing Dato-DXd dose** | 7.214 | 10 | 71% | 4 | 29% | 0 | 0% | 0 | 0% |
| **d. Discontinuing Dato-DXd treatment** | 2.786 | 1 | 7% | 5 | 36% | 8 | 57% | 0 | 0% |
| **e. Other consideration** | 6.667 | 2 | 13% | 1 | 7% | 0 | 0% | 0 | 0% |
| **Treatment approaches for severe OM/S** | | | | | | | | | |
| **23. Based on current guidelines for stomatitis management, I would consider the following treatments for severe stomatitis in patients receiving Dato-DXd treatment in addition to treatments considered for mild or moderate stomatitis:** | | | | | | | | | |
| **a. Systemic oral corticosteroids** | 6.857 | 10 | 71% | 3 | 21% | 1 | 7% | 0 | 0% |
| **b. Daily low-level laser therapy** | 4 | 0 | 0% | 7 | 50% | 4 | 29% | 3 | 21% |
| **c. Systemic opioids** | 7.846 | 12 | 92% | 1 | 8% | 0 | 0% | 0 | 0% |
| **d. Others** | 8 | 1 | 7% | 0 | 0% | 0 | 0% | 0 | 0% |
| **24. For patients experiencing severe stomatitis while receiving Dato-DXd treatment, I would consider:** | | | | | | | | | |
| **a. Keeping the Dato-DXd treatment regimen as is** | 1.429 | 0 | 0% | 0 | 0% | 14 | 100% | 0 | 0% |
| **b. Delaying/interruption Dato-DXd treatment** | 8.357 | 13 | 93% | 0 | 0% | 1 | 7% | 0 | 0% |
| **c. Reducing Dato-DXd dose** | 8 | 12 | 86% | 1 | 7% | 1 | 7% | 0 | 0% |
| **d. Discontinuing Dato-DXd treatment** | 6.429 | 8 | 57% | 5 | 36% | 1 | 7% | 0 | 0% |
| **e. Other** | 9 | 1 | 7% | 0 | 0% | 0 | 0% | 0 | 0% |
| **Section 3: OM/S patient management** | | | | | | | | | |
| **Best practices for managing patients with OM/S** | | | | | | | | | |
| **25. I believe that educating care team members who spend significant time with the patients (e.g., nurses) on the prevention, identification, and management of stomatitis is important** | 8.733 | 15 | 100% | 0 | 0% | 0 | 0% | 0 | 0% |
| **26. I would discuss the patient’s stomatitis presentation and management strategies with the care team prior to stomatitis management initiation** | 8.267 | 14 | 93% | 1 | 7% | 0 | 0% | 0 | 0% |
| **27. When making decisions on the management of stomatitis or potential changes to the Dato-DXd treatment regimen, the patient’s quality of life should be a key consideration** | 8.867 | 15 | 100% | 0 | 0% | 0 | 0% | 0 | 0% |
| **28. Prior to each Dato-DXd infusion, I would ask the patient about their stomatitis prophylaxis / treatment adherence and quality of life to inform if other stomatitis interventions are needed** | 8.857 | 14 | 100% | 0 | 0% | 0 | 0% | 0 | 0% |
| **29. I would evaluate the following patient outcomes while managing patients experiencing stomatitis while receiving Dato-DXd treatment:** | | | | | | | | | |
| **a. Pain level** | 8.8 | 15 | 100% | 0 | 0% | 0 | 0% | 0 | 0% |
| **b. Weight** | 8.6 | 14 | 93% | 1 | 7% | 0 | 0% | 0 | 0% |
| **c. Nutritional status** | 8.4 | 13 | 87% | 2 | 13% | 0 | 0% | 0 | 0% |
| **d. Quality of life** | 8.867 | 15 | 100% | 0 | 0% | 0 | 0% | 0 | 0% |
| **e. Other** | 9 | 2 | 13% | 0 | 0% | 0 | 0% | 0 | 0% |
| **30. Stomatitis management should be adapted to the individual patient due to variability in presentation and differing levels of pain tolerance** | 8.267 | 14 | 93% | 1 | 7% | 0 | 0% | 0 | 0% |
| **31. I would discuss prophylactic and management approaches for managing stomatitis with my patient to determine which approaches they are comfortable with and can access** | 8.333 | 14 | 93% | 0 | 0% | 1 | 7% | 0 | 0% |
| **Management of OM/S with Dato-DXd** | | | | | | | | | |
| **32. Given adequate guidance on the management of stomatitis in patients receiving Dato-DXd treatment, I would consider the risk of stomatitis as a manageable adverse event** | 8 | 14 | 93% | 1 | 7% | 0 | 0% | 0 | 0% |
| **33. Given adequate guidance on the management of stomatitis in patients receiving Dato-DXd treatment, I would be comfortable prescribing Dato-DXd to my patients** | 8.5 | 13 | 93% | 1 | 7% | 0 | 0% | 0 | 0% |
| **34. Based on my clinical experience, I believe stomatitis seen in patients receiving Dato-DXd treatment can be adequately controlled with the preventive and treatment measures outlined in this study** | 7.643 | 12 | 86% | 2 | 14% | 0 | 0% | 0 | 0% |

Legend:

NA = not answered

## Supplementary Table 6 Results from the second survey, summary of scoring

|  | **#agree** | **% agree** | **# of neither nor** | **% neither nor** | **# of disagree** | **% disagree** | **# of NA** | **% of NA** |
| --- | --- | --- | --- | --- | --- | --- | --- | --- |
| **1a. When determining if a patient has stomatitis, a physical examination and patient-reported symptoms are sufficient to make a diagnosis.** | 14 | 100.00% | 0 | 0% | 0 | 0% | 0 | 0% |
| **2. As part of my prophylactic strategy to reduce the likelihood and severity of stomatitis, I would recommend ‘do no harm’ behavioral changes for my patient, including:** | | | | | | | | |
| **a. Teeth brushing -** | 12 | 92.31% | 0 | 0% | 1 | 8% | 0 | 0% |
| **b. Flossing -** | 10 | 76.92% | 2 | 15% | 1 | 8% | 0 | 0% |
| **c. Rinsing their mouth with water** | 11 | 84.62% | 2 | 15% | 0 | 0% | 0 | 0% |
| **d. Avoiding acidic or crunchy foods** | 11 | 84.62% | 2 | 15% | 0 | 0% | 0 | 0% |
| **e. Cryotherapy (ice chips or ice water held in the patient's mouth)** | 11 | 91.67% | 0 | 0% | 1 | 8% | 0 | 0% |
| **f. Rinsing their mouth with a bicarbonate solution** | 11 | 84.62% | 2 | 15% | 0 | 0% | 0 | 0% |
| **6. I believe that only Dato-DXd patients with the following attributes should receive a prophylactic regimen to reduce the likelihood and severity of stomatitis:** | | | | | | | | |
| **Patients with a prior history of mouth sores** | 2 | 14.29% |  |  | 12 | 86% |  |  |
| **Patients aged 65+ years old** | 2 | 14.29% |  |  | 12 | 86% |  |  |
| **Patients with poor nutritional status** | 2 | 14.29% |  |  | 12 | 86% |  |  |
| **Patients with poor oral hygiene or other oral conditions** | 2 | 14.29% |  |  | 12 | 86% |  |  |
| **Patients with a prior history of smoking** | 1 | 7.14% |  |  | 13 | 93% |  |  |
| **All patients starting Dato-DXd** | 14 | 100.00% |  |  | 0 | 0% |  |  |
| **7. For patients I would place on a prophylactic regimen, I would begin prophylaxis:** | | | | | | | | |
| **Prior to Dato-DXd treatment initiation** | 4 | 28.57% |  |  | 10 | 71% |  |  |
| **At Dato-DXd treatment initiation** | 8 | 57.14% |  |  | 6 | 43% |  |  |
| **After Dato-DXd treatment initiation, in patients WITHOUT stomatitis symptoms** | 0 | 0.00% |  |  | 14 | 100% |  |  |
| **After Dato-DXd treatment initiation, in patients WITH stomatitis symptoms** | 2 | 14.29% |  |  | 12 | 86% |  |  |
| **8. As part of my prophylactic and ongoing management strategy of stomatitis, I would recommend a dexamethasone mouth rinse to my Dato-DXd patients** | 12 | 85.71% | 1 | 7% | 1 | 7% | 0 | 0% |
| **9. I would recommend my Dato-DXd patients use the dexamethasone mouth rinse with the following frequency:** | | | | | | | | |
| **1x per day** | 0 | 0.00% |  |  | 14 | 100% |  |  |
| **2x per day** | 5 | 35.71% |  |  | 9 | 64% |  |  |
| **3x per day** | 3 | 21.43% |  |  | 11 | 79% |  |  |
| **4x per day** | 5 | 35.71% |  |  | 9 | 64% |  |  |
| **I would not recommend a dexamethasone mouth rinse to my Dato-DXd patients** | 1 | 7.14% |  |  | 13 | 93% |  |  |
| **10. I would recommend my Dato-DXd patients swish the dexamethasone mouth rinse in their mouth for the following period of time before discarding:** | | | | | | | | |
| **Under 1 minute** | 2 | 14.29% |  |  | 12 | 86% |  |  |
| **1 minute** | 5 | 35.71% |  |  | 9 | 64% |  |  |
| **1-2 minutes** | 4 | 28.57% |  |  | 10 | 71% |  |  |
| **2-3 minutes** | 2 | 14.29% |  |  | 12 | 86% |  |  |
| **I would not recommend a dexamethasone mouth rinse to my Dato-DXd patients** | 1 | 7.14% |  |  | 13 | 93% |  |  |
| **11. If dexamethasone rinse is not available, I would recommend another steroid mouth rinse which does not contain alcohol to my Dato-DXd patients.** | 10 | 76.92% | 2 | 15% | 1 | 8% | 0 | 0% |
| **12. In the absence of a steroid mouth rinse, I would recommend a bland (i.e., non-alcoholic and/or bicarbonate-containing) mouth rinse to my Dato-DXd patients.** | 12 | 85.71% | 1 | 7% | 1 | 7% | 0 | 0% |
| **15. [Breast Cancer Experts only] When determining my stomatitis prevention and treatment strategies for Dato-DXd, I would reference Everolimus guidance for best practices. Guidance includes: Dexamethasone mouth rinse (10 ml, 0.5mg/5ml solution) 4x/day, 2 minutes per time** | 5 | 100.00% | 0 | 0% | 0 | 0% | 0 | 0% |
| **16. For the following Dato-DXd patients, I would monitor for signs of stomatitis at the following frequency: [Please select one option per stomatitis severity]** | | | | | | | | |
| **Non-symptomatic - 1x per treatment cycle (3 weeks)** | 10 | 71.43% |  |  | 4 | 29% |  |  |
| **Non-symptomatic - 1x per every 2 treatment cycles (6 weeks)** | 2 | 14.29% |  |  | 12 | 86% |  |  |
| **Non-symptomatic - Every 6 months** | 0 | 0.00% |  |  | 14 | 100% |  |  |
| **Non-symptomatic - Once a year** | 0 | 0.00% |  |  | 14 | 100% |  |  |
| **Non-symptomatic - I would only monitor once symptoms arise** | 2 | 14.29% |  |  | 12 | 86% |  |  |
| **Mild presentation of stomatitis - 1x per treatment cycle (3 weeks)** | 13 | 92.86% |  |  | 1 | 7% |  |  |
| **Mild presentation of stomatitis - 1x per every 2 treatment cycles (6 weeks)** | 1 | 7.14% |  |  | 13 | 93% |  |  |
| **Mild presentation of stomatitis - Every 6 months** | 0 | 0.00% |  |  | 14 | 100% |  |  |
| **Mild presentation of stomatitis - Once a year** | 0 | 0.00% |  |  | 14 | 100% |  |  |
| **Mild presentation of stomatitis - I would only monitor once symptoms arise** | 0 | 0.00% |  |  | 14 | 100% |  |  |
| **Moderate presentation of stomatitis - 1x per treatment cycle (3 weeks)** | 13 | 92.86% |  |  | 1 | 7% |  |  |
| **Moderate presentation of stomatitis - 1x per every 2 treatment cycles (6 weeks)** | 1 | 7.14% |  |  | 13 | 93% |  |  |
| **Moderate presentation of stomatitis - Every 6 months** | 0 | 0.00% |  |  | 14 | 100% |  |  |
| **Moderate presentation of stomatitis - Once a year** | 0 | 0.00% |  |  | 14 | 100% |  |  |
| **Moderate presentation of stomatitis - I would only monitor once symptoms arise** | 0 | 0.00% |  |  | 14 | 100% |  |  |
| **Severe presentation of stomatitis - 1x per treatment cycle (3 weeks)** | 13 | 92.86% |  |  | 1 | 7% |  |  |
| **Severe presentation of stomatitis - 1x per every 2 treatment cycles (6 weeks)** | 1 | 7.14% |  |  | 13 | 93% |  |  |
| **Severe presentation of stomatitis - Every 6 months** | 0 | 0.00% |  |  | 14 | 100% |  |  |
| **Severe presentation of stomatitis - Once a year** | 0 | 0.00% |  |  | 14 | 100% |  |  |
| **Severe presentation of stomatitis - I would only monitor once symptoms arise** | 0 | 0.00% |  |  | 14 | 100% |  |  |
| **18. I would recommend increasing the prophylactic and on-going stomatitis management strategies and NOT alter Dato-DXd treatment regiment upon seeing the following symptoms:** | | | | | | | | |
| **a. A patient reports mild oral pain, no ulceration, and limited* change in diet needed without any interference in oral intake*Includes avoiding spicy or acidic food** | 12 | 85.71% | 1 | 7% | 1 | 7% | 0 | 0% |
| **b. A patient reports mild oral pain, slight ulceration, and limited* change in diet needed without any interference in oral intake*Includes avoiding spicy or acidic food** | 11 | 78.57% | 3 | 21% | 0 | 0% | 0 | 0% |
| **c. A patient reports mild oral pain, slight ulceration, and some* change in diet needed without any interference in oral intake*Some solid foods may be limited and some change to diet may be required** | 9 | 64.29% | 3 | 21% | 2 | 14% | 0 | 0% |
| **A patient reports moderate oral pain, ulceration, and a moderate change in diet needed without any interference in oral intake** | 6 | 42.86% | 1 | 7% | 7 | 50% | 0 | 0% |
| **e. A patient reports severe oral pain, ulceration, and significant interference with oral intake** | 4 | 28.57% | 1 | 7% | 9 | 64% | 0 | 0% |
| **19. I would consider DELAYING Dato-DXd treatment regimen upon seeing the following symptoms:** | | | | | | | | |
| **a. A patient reports mild oral pain, no ulceration, and limited* change in diet needed without any interference in oral intake*Includes avoiding spicy or acidic food** | 2 | 14.29% | 1 | 7% | 11 | 79% | 0 | 0% |
| **b. A patient reports mild oral pain, slight ulceration, and limited* change in diet needed without any interference in oral intake*Includes avoiding spicy or acidic food** | 2 | 14.29% | 3 | 21% | 9 | 64% | 0 | 0% |
| **c. A patient reports mild oral pain, slight ulceration, and some* change in diet needed without any interference in oral intake*Some solid foods may be limited and some change to diet may be required** | 3 | 21.43% | 4 | 29% | 7 | 50% | 0 | 0% |
| **d. A patient reports moderate oral pain, ulceration, and a moderate change in diet needed without any interference in oral intake** | 10 | 71.43% | 1 | 7% | 3 | 21% | 0 | 0% |
| **e. A patient reports severe oral pain, ulceration, and significant interference with oral intake** | 11 | 91.67% | 0 | 0% | 1 | 8% | 0 | 0% |
| **f. A patient exhibits signs of dehydration** | 10 | 83.33% | 1 | 8% | 1 | 8% | 0 | 0% |
| **g. A patient experiences significant weight loss** | 10 | 83.33% | 1 | 8% | 1 | 8% | 0 | 0% |
| **h. A patient has severe pain and is hospitalized for stabilization and/or pain control** | 9 | 81.82% | 1 | 9% | 1 | 9% | 0 | 0% |
| **20. I would consider REDUCING DOSAGE of Dato-DXd treatment regimen upon seeing the following symptoms:** | | | | | | | | |
| **a. A patient reports mild oral pain, no ulceration, and limited* change in diet needed without any interference in oral intake*Includes avoiding spicy or acidic food** | 2 | 14.29% | 0 | 0% | 12 | 86% | 0 | 0% |
| **b. A patient reports mild oral pain, slight ulceration, and limited* change in diet needed without any interference in oral intake*Includes avoiding spicy or acidic food** | 1 | 7.14% | 2 | 14% | 11 | 79% | 0 | 0% |
| **c. A patient reports mild oral pain, slight ulceration, and some* change in diet needed without any interference in oral intake*Some solid foods may be limited and some change to diet may be required** | 1 | 7.14% | 6 | 43% | 7 | 50% | 0 | 0% |
| **d. A patient reports moderate oral pain, ulceration, and a moderate change in diet needed without any interference in oral intake** | 6 | 54.55% | 2 | 18% | 3 | 27% | 0 | 0% |
| **e. A patient reports severe oral pain, ulceration, and significant interference with oral intake** | 7 | 70.00% | 2 | 20% | 1 | 10% | 0 | 0% |
| **f. A patient exhibits signs of dehydration** | 7 | 77.78% | 1 | 11% | 1 | 11% | 0 | 0% |
| **g. A patient experiences significant weight loss** | 8 | 88.89% | 0 | 0% | 1 | 11% | 0 | 0% |
| **h. A patient has severe pain and is hospitalized for stabilization and/or pain control** | 9 | 81.82% | 1 | 9% | 1 | 9% | 0 | 0% |
| **21. I would consider DISCONTINUATION of the Dato-DXd treatment regimen upon seeing the following symptoms:** | | | | | | | | |
| **a. A patient reports moderate oral pain, ulceration, and a moderate change in diet needed without any interference in oral intake** | 0 | 0.00% | 1 | 8% | 12 | 92% | 0 | 0% |
| **b. A patient reports severe oral pain, ulceration, and significant interference with oral intake** | 5 | 41.67% | 3 | 25% | 4 | 33% | 0 | 0% |
| **c. A patient exhibits signs of dehydration** | 4 | 33.33% | 4 | 33% | 4 | 33% | 0 | 0% |
| **d. A patient experiences significant weight loss** | 6 | 46.15% | 3 | 23% | 3 | 23% | 1 | 8% |
| **e. A patient has severe pain and is hospitalized for stabilization and/or pain control** | 12 | 85.71% | 2 | 14% | 0 | 0% | 0 | 0% |

Legend:

NA = not answered

## References

[1 Major R&D Pipeline: Daiichi Sankyo. Daiichi Sankyo, 2022.](https://www.zotero.org/google-docs/?T1DLEi)

[2 Bardia A. Datopotamab deruxtecan (Dato-DXd) vs chemotherapy in previously-treated inoperable or metastatic hormone receptor-positive, HER2-negative (HR+/HER2–) breast cancer: Primary results from the randomised Phase 3 TROPION-Breast01 trial. 2023.](https://www.zotero.org/google-docs/?T1DLEi)

[3 Lisberg A. Datopotamab deruxtecan (Dato-DXd) vs docetaxel in previously treated advanced/metastatic (adv/met) non-small cell lung cancer (NSCLC): Results of the randomized phase 3 study TROPION-Lung01. 2023.](https://www.zotero.org/google-docs/?T1DLEi)

[4 Heist R. Clinical management, monitoring, and prophylaxis of adverse events of special interest associated with datopotamab deruxtecan. *Cancer Treatment Reviews* 2024; **125**.](https://www.zotero.org/google-docs/?T1DLEi)

[5 Negrin R, Treister N. Oral toxicity associated with systemic anticancer therapy. .](https://www.zotero.org/google-docs/?T1DLEi)

[6 Amiri Khosroshahi R. Nutritional interventions for the prevention and treatment of cancer therapy-induced oral mucositis: an umbrella review of systematic reviews and meta-analysis. *Nutr Rev* 2023; **nuac105**. DOI:10.1093/nutrit/nuac105.](https://www.zotero.org/google-docs/?T1DLEi)

[7 Elad S. MASCC/ISOO clinical practice guidelines for the management of mucositis secondary to cancer therapy. *Cancer* 2020; **126**: 4423–31.](https://www.zotero.org/google-docs/?T1DLEi)

[8 Pulito C. Oral mucositis: the hidden side of cancer therapy. *J Exp Clin Cancer Res* 2020; **39**: 210.](https://www.zotero.org/google-docs/?T1DLEi)

[9 Suzuki A. Management of cancer treatment-induced oral mucositis. In: Inflammation and Oral Cancer. Elsevier, 2022: 183–97.](https://www.zotero.org/google-docs/?T1DLEi)

[10 Vogel WH, Jennifer P. Management Strategies for Adverse Events Associated With EGFR TKIs in Non-Small Cell Lung Cancer. *J Adv Pract Oncol* 2016; **7**: 723–35.](https://www.zotero.org/google-docs/?T1DLEi)

[11 Elad S, Yarom N, Zadik Y, Kuten-Shorrer M, Sonis ST. The broadening scope of oral mucositis and oral ulcerative mucosal toxicities of anticancer therapies. *CA Cancer J Clin* 2022; **72**: 57–77.](https://www.zotero.org/google-docs/?T1DLEi)

[12 Jasiewicz F, Qurban Z, Hughes C. Treatment-induced mucositis in oncology. *Br J Hosp Med (Lond* 2022; **83**: 1–8.](https://www.zotero.org/google-docs/?T1DLEi)

[13 Kwon Y. Mechanism-based management for mucositis: option for treating side effects without compromising the efficacy of cancer therapy. *Onco Targets Ther* 2016; **9**: 2007–16.](https://www.zotero.org/google-docs/?T1DLEi)

[14 Califano R. Expert Consensus on the Management of Adverse Events from EGFR Tyrosine Kinase Inhibitors in the UK. *Drugs* 2015; **75**: 1335–48.](https://www.zotero.org/google-docs/?T1DLEi)

[15 Edwards RL. Afatinib Therapy: Practical Management of Adverse Events With an Oral Agent for Non-Small Cell Lung Cancer Treatment. *Clin J Oncol Nurs* 2018; **22**: 542–8.](https://www.zotero.org/google-docs/?T1DLEi)

[16 Thomas CM. Mucositis in Cancer Patients: A Review. *US Pharm* 2016; **41**: 6–8.](https://www.zotero.org/google-docs/?T1DLEi)

[17 Kusiak A, Jereczek-Fossa BA, Cichońska D, Alterio D. Oncological-Therapy Related Oral Mucositis as an Interdisciplinary Problem-Literature Review. *Int J Environ Res Public Health* 2020; **17**: 2464.](https://www.zotero.org/google-docs/?T1DLEi)

[18 Chambers MS, Rugo HS, Litton JK, Meiller TF. Stomatitis associated with mammalian target of rapamycin inhibition: A review of pathogenesis, prevention, treatment, and clinical implications for oral practice in metastatic breast cancer. *J Am Dent Assoc* 2018; **149**: 291–8.](https://www.zotero.org/google-docs/?T1DLEi)

[19 Calvo AS, Rochefort J, Javelot MJ, Descroix V, Lescaille G. Management of mTOR inhibitors oral mucositis: current state of knowledge. *J Oral Med Oral Surg* 2019; **25**: 11.](https://www.zotero.org/google-docs/?T1DLEi)

[20 Chaveli-López B, Bagán-Sebastián JV. Treatment of oral mucositis due to chemotherapy. *J Clin Exp Dent* 2016; **8**: 201–9.](https://www.zotero.org/google-docs/?T1DLEi)
